# Biosynthesis of natural and halogenated plant monoterpene indole alkaloids in yeast

**DOI:** 10.1038/s41589-023-01430-2

**Published:** 2023-11-06

**Authors:** Samuel A. Bradley, Beata J. Lehka, Frederik G. Hansson, Khem B. Adhikari, Daniela Rago, Paulina Rubaszka, Ahmad K. Haidar, Ling Chen, Lea G. Hansen, Olga Gudich, Konstantina Giannakou, Bettina Lengger, Ryan T. Gill, Yoko Nakamura, Thomas Dugé de Bernonville, Konstantinos Koudounas, David Romero-Suarez, Ling Ding, Yijun Qiao, Thomas M. Frimurer, Anja A. Petersen, Sébastien Besseau, Sandeep Kumar, Nicolas Gautron, Celine Melin, Jillian Marc, Remi Jeanneau, Sarah E. O’Connor, Vincent Courdavault, Jay D. Keasling, Jie Zhang, Michael K. Jensen

**Affiliations:** 1 Novo Nordisk Foundation Center for Biosustainability, Technical University of Denmark, Lyngby, Denmark; 2 Department of Natural Product Biosynthesis, Max Planck Institute for Chemical Ecology, Jena, Germany; 3 EA2106 Biomolécules et Biotechnologies Végétales, Université de Tours, Tours, France; 4 Department of Bioengineering, Technical University of Denmark, Lyngby, Denmark; 5 Novo Nordisk Foundation Center for Basic Metabolic Research, University of Copenhagen, Copenhagen, Denmark; 6 Axyntis, Pithiviers, France; 7 Joint BioEnergy Institute, Emeryville, CA USA; 8 Biological Systems and Engineering Division, Lawrence Berkeley National Laboratory, Berkeley, CA USA; 9 Department of Chemical and Biomolecular Engineering, University of California, Berkeley, Berkeley, CA USA; 10 Department of Bioengineering, University of California, Berkeley, Berkeley, CA USA; 11 Center for Synthetic Biochemistry, Institute for Synthetic Biology, Shenzhen Institutes of Advanced Technologies, Shenzhen, China

**Keywords:** Chemical modification, Natural products, Metabolic pathways

## Abstract

Monoterpenoid indole alkaloids (MIAs) represent a large class of plant natural products with marketed pharmaceutical activities against a wide range of indications, including cancer, malaria and hypertension. Halogenated MIAs have shown improved pharmaceutical properties; however, synthesis of new-to-nature halogenated MIAs remains a challenge. Here we demonstrate a platform for de novo biosynthesis of two MIAs, serpentine and alstonine, in baker’s yeast *Saccharomyces cerevisiae* and deploy it to systematically explore the biocatalytic potential of refactored MIA pathways for the production of halogenated MIAs. From this, we demonstrate conversion of individual haloindole derivatives to a total of 19 different new-to-nature haloserpentine and haloalstonine analogs. Furthermore, by process optimization and heterologous expression of a modified halogenase in the microbial MIA platform, we document de novo halogenation and biosynthesis of chloroalstonine. Together, this study highlights a microbial platform for enzymatic exploration and production of complex natural and new-to-nature MIAs with therapeutic potential.

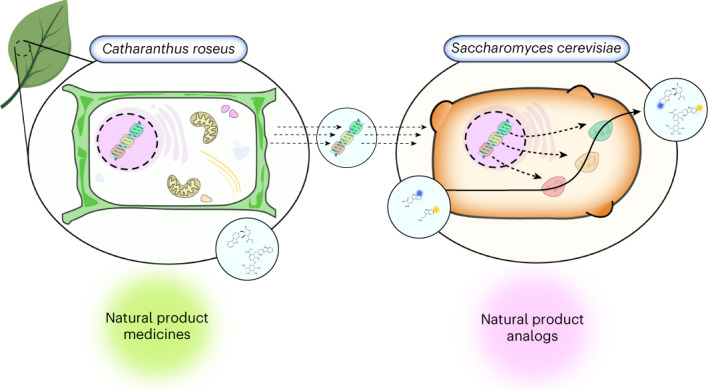

## Abstract (web summary)

A yeast platform for de novo biosynthesis of medicinal plant compounds has now been reported. The platform was used to explore the biocatalytic potential of refactored plant pathways and resulted in the production of 19 halogenated derivatives with therapeutic potential.

## Main

Monoterpenoid indole alkaloids (MIAs) are an important group of plant secondary metabolites that possess various medicinal properties, including marketed chemotherapeutic agents such as vinblastine and vincristine and the antiarrhythmic drug ajmaline^1^. Likewise, numerous bioactive MIAs are used to treat human illnesses outside the clinic, including ibogaine for the treatment of opioid use disorder and withdrawal symptoms^2^ and the stereoisomers alstonine (**1a**) and serpentine (**2a**) with reported therapeutic effects against a broad number of indications, such as psychotic disorders, cancer and malaria^3^. Beyond the range of naturally occurring bioactive MIAs, unnatural analogs with improved potency have also been reported^4^.

Producing MIAs at scale for medicinal use is challenging. Source extraction from plants can suffer from supply chain shortages and generally poor yields^5^, whereas total chemical synthesis is plagued by difficulties separating stereoisomers^6^.

A biotechnological solution for the production of both natural and modified MIAs is microbial cell factories, which are engineered to produce bioactive phytochemicals using fermentation. Indeed, heterologous expression of plant genes in baker’s yeast *Saccharomyces cerevisiae* has facilitated the production of various plant-derived pharmaceuticals, including MIAs^7–9^. Furthermore, valorization of commercially available precursors to downstream MIA-based active pharmaceutical ingredients has been demonstrated^10^, while Liu et al. recently documented the de novo production of a complex class of heteroyohimbines, including tetrahydroalstonine (**3a**) and ajmalicine (**4a**)^11^, which are the direct precursors for the MIA enantiomers alstonine (**1a**) and serpentine (**2a**)^12^. With continued advancements in synthetic biology, metabolic engineering and plant biosynthetic pathway discoveries^13^, refactoring de novo production of bioactive MIAs in microbial cells is now facilitated.

In addition to the interest in fermentation-based de novo production of MIAs from simple feedstocks (for example, glucose and amino acids), several pioneering studies have investigated enzymatic production of unnatural MIAs from unnatural substrate analogs in vitro^14^ and in planta^15^ by feeding unnatural precursor analogs. Early investigations into the promiscuity of individual enzymes were followed by heterologous enzyme expression for directly introducing unnatural elements into MIA precursors^16^. In a landmark study, two tryptophan halogenases from *Lechevalieria aerocolonigenes* (*Lae*RebH) and *Streptomyces rugosporus* (*Sru*PyrH) were expressed in the MIA-producing plant *Catharanthus roseus*, resulting in de novo production of chlorinated and brominated MIAs. Yet, as these new-to-nature chemical spaces include both regioselective considerations and choice of halogen, it remains a challenge to mitigate barriers within new-to-nature chemistries in slow-growing plants with limited genetic tractability. Likewise, regioselective chemical halogenation of pharmacophores and total chemical synthesis are complicated due to the numerous stereocenters often found in natural products, not to mention the difficulties of scaling up production once a lead has been identified^17^. Here, the advanced genetic toolbox, short generation times and amenable metabolism of *S. cerevisiae* for de novo production of strictosidine (**5a**) and heteroyohimbine MIAs^9,11^ makes this chassis ripe for systematically exploring and prototyping whole-cell biocatalysis of new-to-nature MIA chemistries.

In this work, we report the engineering of yeasts for de novo production of bioactive alstonine (**1a**) and serpentine (**2a**) and demonstrate their utility for exploring enzyme promiscuity and metabolic limitations in refactored biosynthetic pathways while producing MIA derivatives. Ultimately, this approach enabled the biosynthesis of 19 halogenated heteroyohimbines (**1a–i** and **2a–l**) and de novo biosynthesis of chloroalstonine (**1f**).

## Results

### De novo alstonine and serpentine biosynthesis in yeast

**Fig. 1:**
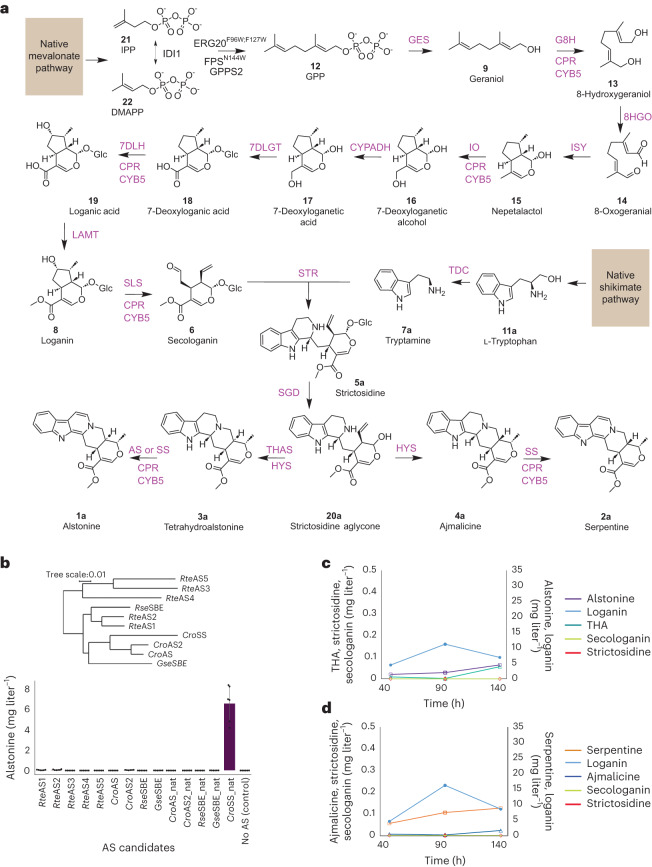
De novo alstonine and serpentine production in yeast. **a**, Integration of plant biosynthetic pathways with native yeast metabolic pathways to produce alstonine and serpentine. IPP, isopentenyl pyrophosphate; DMAPP, dimethylallyl pyrophosphate; GPPS, GPP synthase; FPS^N144W^, FPP synthase N144W variant; CPR, NADPH-cytochrome P450 reductase; CYB5, cytochrome b5; GES, geraniol synthase; G8H, geraniol 8-hydroxylase; 8HGO, 8-hydroxygeraniol oxidoreductase; ISY, iridoid synthase; IO, iridoid oxidase; CYPADH, alcohol dehydrogenase 2; 7DLGT, 7-deoxyloganetic acid glucosyl transferase; 7DLH, 7-deoxyloganic acid hydroxylase; LAMT, loganic acid O-methyltransferase; TDC, tryptophan decarboxylase; SLS, secologanin synthase; STR, strictosidine synthase. **b**, Screen of AS candidates in YPD cultivation medium. Gene candidates are linked to strain identifiers as follows: *Rte*AS1 (Sc87), *Rte*AS2 (Sc88), *Rte*AS3 (Sc90), *Rte*AS4 (Sc92), *Rte*AS5 (Sc94), *Cro*AS_nat (Sc96), *Rse*SBE_nat (Sc97), *Gse*SBE_nat (Sc98), *Cro*AS2_nat (Sc100), *Cro*AS2 (Sc101), *Cro*AS (Sc102), *Rse*SBE (Sc103), *Gse*SBE (Sc104) and *Cro*SS_nat (Sc157) and a negative-control strain (Sc86). **c**,**d**, Representative production profiles for alstonine (**c**), serpentine (**d**) and pathway intermediates using a small-scale fed-batch process for strains Sc112 and Sc85, respectively, cultivated in 1 ml of 3× SC medium supplemented with 3 mM tryptophan. For **b**, *n* = 3, and error bars represent 1 s.d. from the mean, with data points overlaid as black dots. Source data

**Extended Data Fig. 1:**
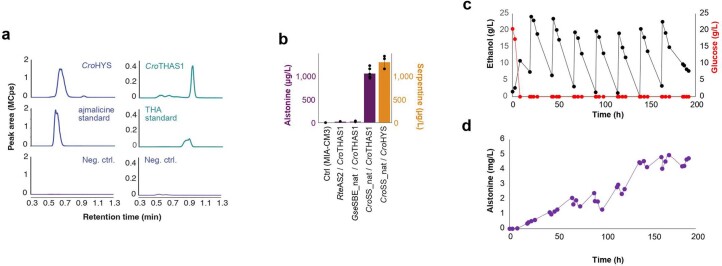
*De novo* alstonine and serpentine production in yeast. **a**. Representative chromatogram from LC-MS analysis of spent medium from yeast (MIA-CM-3) expressing *Cro*THAS1+*Rse*SGD and *Cro*HYS+*Rse*SGD when cultivated in synthetic complete (SC) medium supplemented with secologanin and tryptamine. Controls include tetrahydroalstonine and ajmalicine standards, as well as a negative control (MIA-CM-3). **b**. *De novo* production of alstonine produced by *Cro*THAS1+*Rte*AS2 (Sc77), *Cro*THAS1+*Gse*SBE_nat (Sc78), and *Cro*THAS1+*Cro*SS_nat (Sc112), and *de novo* production of serpentine by yeast strain expressing *Cro*HYS+*Cro*SS_nat (Sc85), when cultivated in 3 x SC medium supplemented with 3 mM tryptophan. The strains are based on MIA-CM-3, with a genome integrated copy of *Rse*SGD. **c, d**. Carbon feedstock (**c**) and alstonine (**d**) levels during the 20 h glucose batch followed by a pulsed-ethanol fed-batch process of *de novo* alstonine-producing strain ScH144 in 2 ltr bioreactor. For b, n = 3 and error bars represent one standard deviation from the mean. Source data

In plants, conversion of strictosidine (**5a**) to alstonine (**1a**) or serpentine (**2a**) is catalyzed by strictosidine-β-d-glucosidase (SGD)^18^, the dehydrogenases tetrahydroalstonine synthase (THAS)^19^ or heteroyohimbine synthase (HYS)^20^ and the recently discovered cytochrome P450 enzymes alstonine synthase (AS)^21^ and serpentine synthase (SS^12^; Fig. 1a). For the establishment of a microbial platform for producing alstonine (**1a**) and serpentine (**2a**), we initially characterized the promiscuity of the two heteroyohimbine dehydrogenases *C. roseus* THAS1 (*Cro*THAS1) and *C. roseus* HYS (*Cro*HYS) in yeast. These enzymes were expressed in a de novo strictosidine-producing strain (MIA-CM-3 (ref. ^9^); Supplementary Table 1) together with SGD from *Rauvolfia serpentina* (*Rse*SGD; Fig. 1a). Here, yeast expressing *Rse*SGD and *Cro*THAS1 produced tetrahydroalstonine (THA), whereas yeast expressing *Rse*SGD and *Cro*HYS produced ajmalicine (**4a**) and a small amount of THA (**3a**), as also recently reported (Extended Data Fig. 1a)^11^. Next, for the characterization of P450 enzymes catalyzing the conversion of THA (**3a**) to alstonine (**1a**), we selected candidates reported in the literature, *R. serpentina* sarpagan bridge enzyme (*Rse*SBE), *Gelsemium sempervirens* SBE (*Gse*SBE), *C. roseus* AS (*Cro*AS)^21^ and the recently identified *C. roseus* SS (*Cro*SS)^12^ due to its close homology with *Cro*AS (Fig. 1b). We also mined the *C. roseus* genome for alternative AS candidates^22^. This led to the identification of a sequence displaying 87% identity with *Cro*AS, tentatively named *Cro*AS2 (ref. ^21^). Additionally, we generated a de novo assembled transcriptome of *R. tetraphylla* to search for additional AS candidates. Mining this new resource allowed the identification of two hypothetical P450 enzymes (R1n_CGCTCATT-TATAGCCT_DN432_c0_g1_i4.p1 and BP5_GAGATTCC-GGCTCTGA_DN4992_c0_g1_i3.p1), which displayed more than 75% identity with already characterized AS^21^. Based on this significant identity, these two sequences were tentatively named *Rte*AS1 and *Rte*AS2. Three additional sequences of lower identity were also retained and hereafter named *Rte*AS3 (R1n_CGCTCATT-TATAGCCT_DN14548_c0_g1_i1.p1), *Rte*AS4 (DL4_GAGATTCC-TATAGCCT_DN2810_c0_g2_i3.p1) and *Rte*AS5 (BL2_CGCTCATT-CCTATCCT_DN6055_c0_g1_i1.p1; Fig. 1b).

**Extended Data Fig. 2:**
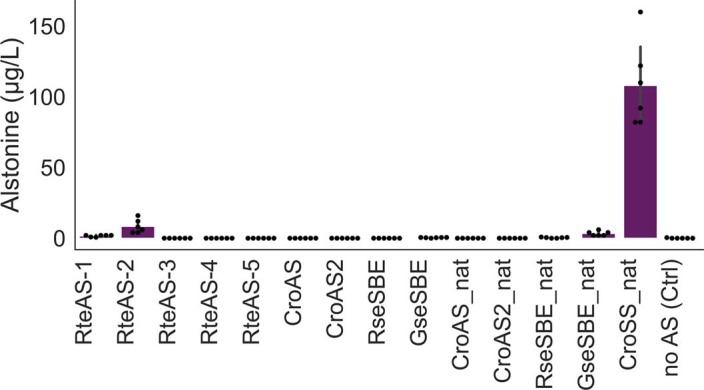
Alstonine titers in yeast strains expressing individual alstonine synthase variants when cultivated in deep-well plates. Titers of alstonine (μg/L) after 144 h cultivations of 6 biological replicates of strains Sc138-153 with 1 mM tryptamine and 0.2 mM secologanin in SC media, + 1 mM Trp. Gene candidates are linked to strain identifiers as follows: RteAS1 (Sc87), RteAS2 (Sc88), RteAS3 (Sc90), RteAS4 (Sc92), RteAS5 (Sc94), CroAS_nat (Sc96), RseSBE_nat (Sc97), GseSBE_nat (Sc98), CroAS2_nat (Sc100), CroAS2 (Sc101), CroAS (Sc102), RseSBE (Sc103), GseSBE (Sc104), CroSS_nat (Sc157) as well as negative control strain (Sc86). The background strain for this screen was MIA-B0, with an integrated pathway from tryptamine and secologanin to THA, allowing for feeding of these close precursors. Mean, n = 6, with error bars indicating SD. Source data

Next, to characterize the catalytic activity of the identified P450 candidates, we used a feeding strategy of secologanin (**6**) and tryptamine (**7a**) to the engineered background strain expressing *C. roseus* cytochrome P450 reductase (*Cro*CPR), *C. roseus* strictosidine synthase (*Cro*STR) and *Rse*SGD and *Cro*THAS1 (Sc86; Fig. 1a). Strains expressing individual P450 candidates were cultivated in synthetic complete (SC) or rich cultivation (yeast extract peptone dextrose (YPD)) medium supplemented with secologanin (**6**) and tryptamine (**7a**; Fig. 1b, Extended Data Fig. 2 and Supplementary Table 1). In this screen, we identified five enzymes producing alstonine (**1a**), namely two synthases from *R. tetraphylla* (*Rte*AS1 and *Rte*AS2 in strains Sc87 and Sc88), SBE from *G. sempervirens* (*Gse*SBE_nat in strain Sc98 or *Gse*SBE in strain Sc104), *Cro*AS2 (strain Sc101) and *Cro*SS (strain Sc157). In YPD medium, we observed up to 20-fold improvements compared to SC medium (Extended Data Fig. 2 and Supplementary Table 1), with *Rte*AS2 (strain Sc88) enabling the production of 71.7 ± 41 µg liter^–1^ and *Cro*SS enabling the production of 6,641 ± 1,757 µg liter^–1^ in YPD (Fig. 1b).

Based on the screen for ASs (Fig. 1b), individual genes encoding *Rte*AS2, *Gse*SBE_nat and *Cro*SS_nat were genomically integrated into the strictosidine-producing strain MIA-CM-3 (ref. ^9^) together with *Rse*SGD and *Cro*THAS1 to test de novo alstonine (**1a**) production (resulting in strains Sc77, Sc78 and Sc112, respectively) or together with *Rse*SGD and *Cro*HYS to test de novo serpentine (**2a**) production (resulting in strain Sc85). All strains were cultivated in a previously optimized medium for small-scale MIA production (3× SC)^9^, in which Sc77 outperformed Sc78 with an alstonine (**1a**) titer of 29.7 ± 4 µg liter^–1^, Sc112 expressing *Cro*SS_nat yielded a further >40-fold increase in alstonine (**1a**) titer of 1,034 ± 94 µg liter^–1^, and Sc85 was observed to produce 1,270 ± 130 µg liter^–1^ serpentine (**2a**). Importantly, although strictosidine (**5a**) was consumed in all strains, THA (**3a**) and ajmalicine (**4a**) accumulated up to 990 ± 192 µg liter^–1^ and 914 ± 3 µg liter^–1^, respectively, in strains Sc77 and Sc78, whereas conversion of THA to alstonine by *Cro*SS_nat in strain Sc112 lowered THA levels to 166 ± 35 µg liter^–1^ (Extended Data Fig. 1b and Supplementary Fig. 1).

To further investigate the physiology and productivities of de novo alstonine (**1a**) and serpentine (**2a**) strains, fed-batch cultivation of strains Sc85 and Sc112 were cultivated in 3× SC medium using controlled microbioreactors (Methods). To prevent accumulation of by-products (for example, ethanol) and improve biomass and product yield, cells were grown in batch culture for 20 h, followed by exponential feeding for 124 h. The highest final titers reached 8.85 mg liter^–1^ serpentine (**2a**) from strain Sc85 and 4.48 mg liter^–1^ alstonine (**1a**) from strain Sc112 (Fig. 1c,d, Table 1 and Supplementary Fig. 2). For this process, we furthermore observed lowered accumulation of pathway intermediates compared to batch cultivation, except for tryptamine (**7a**) and loganin (**8**; Fig. 1c,d and Supplementary Figs. 2–4). Here, tryptamine (**7a**) accumulated to 139 mg liter^–1^ for Sc85 and 162 mg liter^–1^ for Sc112, and loganin (**8**) accumulated to 8.5 mg liter^–1^ for Sc85 and 6.9 mg liter^–1^ for Sc112, indicating the P450 *C. roseus* secologanin synthase (*Cro*SLS) as a bottleneck for MIA production in yeast (Supplementary Fig. 3).

**Table 1:** Fed-batch bioprocess for strains producing serpentine and alstonine

Strain, colony	Biomass (g liter^–1^)^a^	Yield biomass (g per g glucose)	Yield product (mg per g glucose)
Sc85, F1	8.20	0.13	0.14
Sc85, F2	6.60	0.11	0.12
Sc85, D5	5.20	0.08	0.09
Sc112, D2	8.80	0.14	0.07
Sc112, C1	11.00	0.18	0.06
Sc112, D1	10.60	0.17	0.05

Final biomass, yield of biomass and product yields for serpentine produced by Sc85 and alstonine produced by Sc112 during 144 h of cultivation. Data for three different colonies are shown for each of the two production strains.

^a^Biomass was measured using grams (dry weight).

**Extended Data Fig. 3:**
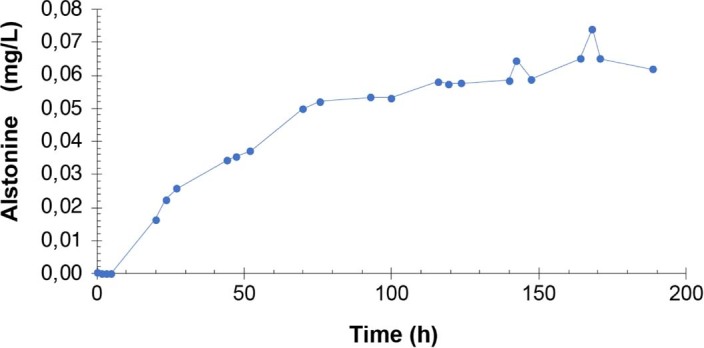
Alstonine production of strain Sc112 in 2 ltr bioreactor. Titers of alstonine (mg/L) during 144 h cultivation in 2 ltr bioreactor with 60 g/ltr glucose feeding. Single measurements. Source data

**Extended Data Fig. 4:**
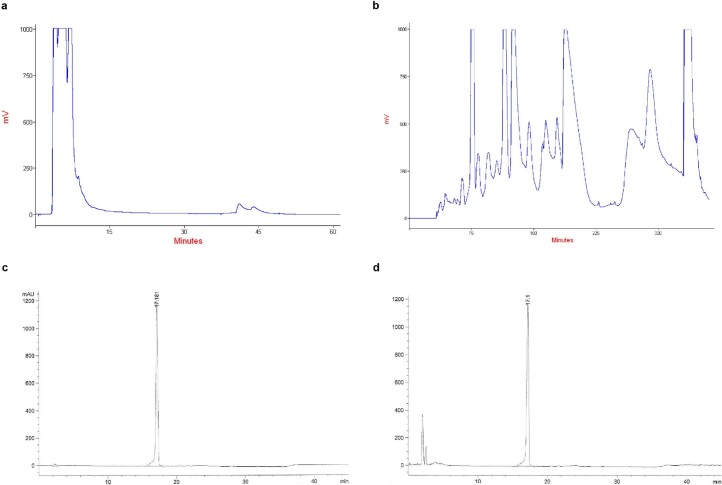
Chromatograms from alstonine purification. **a**. Chromatogram of alstonine purification on DAC LC50 system. Alstonine compound is eluted with a retention time of about 40 min after using washing solvent. **b**. Chromatogram of alstonine purification on a semi-preparative system. Alstonine compound is eluted with a retention time of 332 min after using washing solvent. **c**. Chromatogram of purified alstonine at 250 nm. **d**. Maxplot chromatogram of purified alstonine.

**Extended Data Fig. 5:**
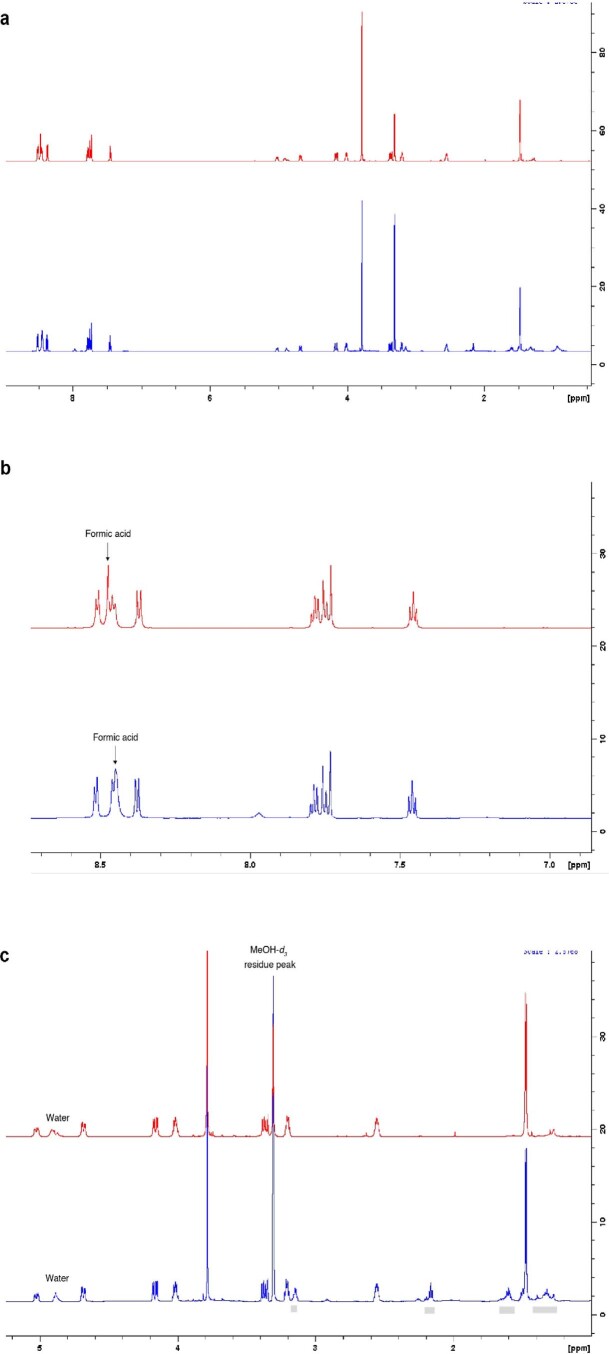
NMR spectra for alstonine produced in yeast. **a**. ^1^H NMR with water suppression full range in MeOH-*d*_*3*_ alstonine standard (red), and SPE purified alstonine from alstonine-producing yeast (blue). **b**. ^1^H NMR with water suppression aromatic range in MeOH-*d*_*3*_ alstonine standard (red), and SPE purified alstonine from enzymatic experiment. **c**. ^1^H NMR with water suppression aliphatic range in MeOH-*d*_*3*_ alstonine standard (red), and SPE purified alstonine from enzymatic experiment. Gray bars indicate impurities.

**Extended Data Fig. 6:**
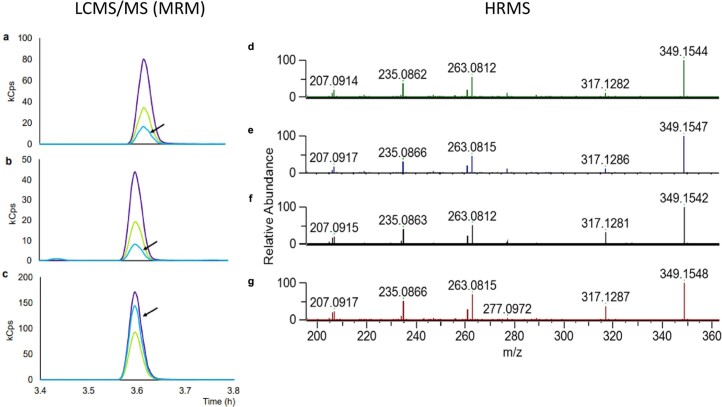
Comparison of alstonine and serpentine standards and producers. **a**–**c**, LCMS analysis of Profile of MS/MS transitions for alstonine standard (**a**), broth of alstonine producer Sc77 (**b**) and serpentine standard (**c**). Transitions characteristic for alstonine and serpentine: 262.9 (gray), 316.9 (blue), and 234.9 (green). **d**–**g**, HRMS analysis of alstonine standard (**d**), broth of alstonine producer Sc112 (**e**), serpentine standard (**f**), and broth of serpentine producer Sc85 (**g**). Source data

Last, the fed-batch bioprocess for Sc112 was scaled up to 2-liter bioreactors. Here, alstonine (**1a**) production was critically affected by this change of scale, with titers below 0.1 mg liter^–1^ (Extended Data Fig. 3). Acknowledging the reported positive contribution to P450-mediated biocatalysis^23^, we decided to delete *ROX1*, a gene encoding a repressor of the heme biosynthetic gene *HEM13*, in Sc112 yielding strain ScH144. In microbioreactor fermentations, ScH144 produced almost 60% more alstonine (**1a**) than Sc112 (16.7 ± 1.33 versus 9.9 ± 0.13 mg liter^–1^) when medium was supplemented with bovine peptone (Supplementary Fig. 5)^11^. Strain ScH144 was fed under a pulsed strategy instead of an exponential strategy given the better production yield observed on glucose during the batch phase in the 2-liter bioreactor with the parental strain Sc112 (1.7 versus 0.8 µg g^–1^). Interestingly, this restored alstonine (**1a**) biosynthesis, reaching 3.6 mg liter^–1^ after 185 h (Supplementary Fig. 6). In agreement with the positive effect of ethanol substrate on terpene synthesis previously reported for geraniol (**9**) and sesquiterpenes, we finally replaced glucose by ethanol as a yeast carbon source^24^. Pulsing ethanol to ScH144 during fed-batch phase in the 2-liter bioreactor further increased alstonine titer (up 4.9 mg liter^–1^), thus reaching the concentration obtained in the microbioreactor with Sc112 (Fig. 1c,d and Extended Data Fig. 1c). Using the spent medium, we performed a four-step purification process (Methods) and purified 2 mg of alstonine (**1a**) at 95% purity (Extended Data Fig. 4). Last, purified alstonine (**1a**; 0.3 mg) was analyzed by ^1^H NMR to validate the structure of the isolated product compared to an authentic alstonine (**1a**) standard, corroborating mass spectrometry (MS) data from broth (Extended Data Figs. 5 and 6).

Taken together, combinatorial engineering of heteroyohimbine biosynthetic pathways in yeast combined with scalable bioprocess optimization enabled de novo production of MIAs at the milligram scale.

### Bioactivity of alstonine

Heteroyohimbines, such as yohimbine and rauwolscine, have been reported as ligands of monoaminergic and serotonergic G-protein-coupled receptor (GPCR) drug targets^17^. Furthermore, the anxiolytic properties of alstonine (**1a**) have been shown to be reverted in mice pretreated with a 5-HT_2A_/5-HT_2C_ antagonist^25^.

**Fig. 2:**
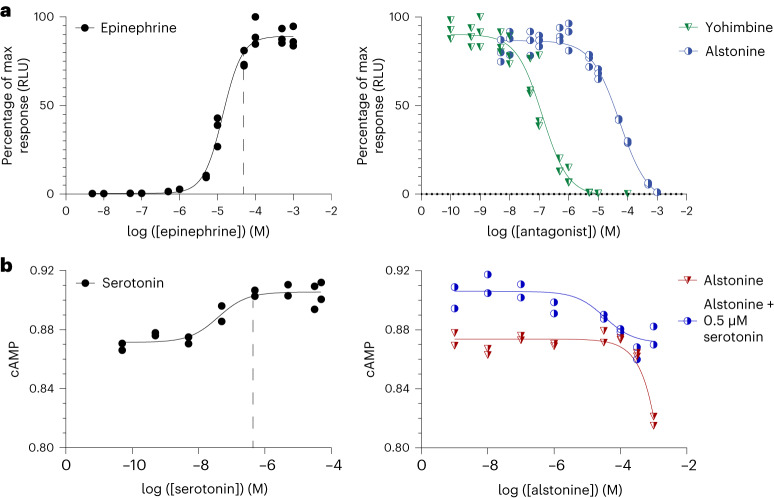
Bioactivity testing of alstonine in yeast and mammalian cells. **a**, Dose–response curves of ADRA2A with the agonist epinephrine (left; *R*^2^ = 0.988) and antagonist activities of yohimbine and alstonine (right; *R*^2^ = 0.965 and 0.980, respectively). Data are reported in relative luminescence units (RLU) based on nano-luciferase (NanoLuc) readouts normalized to the maximum observed luminescence. Data report triplicate biological replicates (*n* = 3) measured on yeast cells heterologously expressing the ADRA2A receptor; [epinephrine], concentration of epinephrine; [antagonist], concentration of antagonist; [serotonin], concentration of serotonin; [alstonine], concentration of alstonine. **b**, Dose–response curves of 5-HT_2C_ with the agonist serotonin (left; *R*^2^ = 0.892) and alstonine with and without 0.5 µM competing serotonin (right; *R*^2^ = 0.792 and 0.913). cAMP levels were monitored using BRET in COS7 cells, and data are reported for biological duplicates (*n* = 2). For all data presented, each replicate is shown, and a non-linear regression model was applied using the least squares method. The dashed lines in **a** and **b** (left) indicate 50 µM and 0.5 µM, respectively, which were the agonist concentrations used in the respective competitor assays (right). Source data

To assess the potential bioactivity of alstonine (**1a**), we first adopted a previously developed antagonist assay against the epinephrine-activated ADRA2A GPCR expressed in yeast cells^26^. To do so, we first estimated the half-maximum effective concentration of epinephrine against the ADRA2A receptor expressed in yeast cells as 14 ± 2 µM, with a 95% confidence interval (CI_95_) of 11.5–16.6 µM (Fig. 2a). Next, we co-incubated ADRA2A-expressing yeast cells (Sc272) with 50 µM epinephrine and different dosages of the known ADRA2A antagonist yohimbine^17^ or alstonine (**1a**). We found that alstonine is a weak antagonist of ADRA2A with a half-maximal inhibitory concentration (IC_50_) of 59 ± 20 µM (CI_95_ of 39.0–79.3 µM), whereas yohimbine exerted an IC_50_ of 0.13 ± 0.04 µM (CI_95_ of 0.089–0.181 µM) against ADRA2A (Fig. 2a). Next, based on the possible link between the anxiolytic effects of alstonine and the 5-HT_2A_/5-HT_2C_ GPCRs in rodents^25^, we were interested to further explore possible direct drug targets of alstonine. As the 5-HT_2_ GPCRs have not been functionalized in yeast^27^, we expressed 5-HT_2C_ in mammalian COS7 cells and tested alstonine activity in the presence and absence of the cognate 5-HT_2C_ agonist serotonin. Here, we observed alstonine as a weak antagonist of the 5-HT_2C_ receptor, albeit no IC_50_ could be reported for the concentration range available (Fig. 2b).

In summary, bioactivity testing of purified alstonine (**1a**) from fermentation-based manufacturing identifies alstonine as a possible antagonist of ADRA2A and 5-HT_2C_ GPCRs.

### De novo biosynthesis of new-to-nature heteroyohimbine in yeast

Motivated by in vitro^14^ and in planta^15^ studies documenting halogenation of MIA precursors and the approved drugs founded on halogenated MIA scaffolds^28^, we aimed to harness the successful refactoring of MIA biosynthetic pathways to systematically investigate the potential for production of new-to-nature MIA analogs in yeast with an initial focus on alstonine (**1a**).

**Extended Data Fig. 7:**
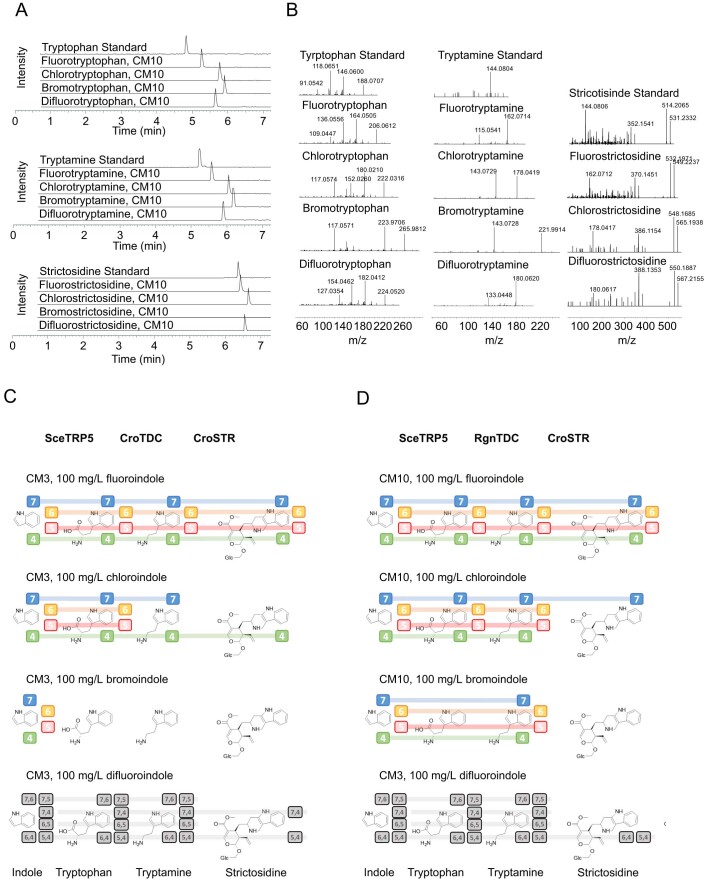
Biosynthesis of halo-strictosidine by MIA-CM3 and MIA-CM10. MIA-CM3 and MIA-CM10 were cultivated (see Methods) with 100 mg/L haloindole and 0.25 mM secologanin and the broth at 144 hours analyzed using HRMS. **a**. Representative chromatograms for MIA standards and fluorinated, chlorinated, brominated and difluorinated derivatives after feeding the corresponding haloindole. **b**. Representative MS-MS spectra extracted from the corresponding peaks in panel a. **c**,**d**, Indole to strictosidine pathways expressed in MIA-CM3 (**c**) and MIA-CM10 (**d**) with the detection of halogenated derivatives overlaid. The presence of a colored or gray box indicates identification of the derivative with a halo-substitution(s) at the indicated carbon (indole numbering). Enzyme abbreviation: TRP5 - tryptophan synthase 5, TDC - tryptophan synthase, STR - strictosidine synthase. Source data

Anticipating a derivative bottleneck at the *C. roseus* tryptophan decarboxylase (*Cro*TDC)-catalyzed step^16^, we initially replaced this enzyme from the de novo strictosidine-producing strain MIA-CM-3 (ref. ^9^) with a more promiscuous homolog from *Ruminococcus gnavus* (*Rgn*TDC^29^; MIA-CM-10). Comparing the ability of MIA-CM-3 and MIA-CM-10 to convert a panel of 18 fluorinated, chlorinated and brominated indoles (**10b–10s**) into halogenated strictosidine (**5b–5n**) revealed that the native tryptophan synthase (encoded by *TRP5*) can combine all indole tested derivatives with serine (Extended Data Fig. 7a,b). Although the bromotryptophan (**11j–11m**) spectra did not correlate with that of the tryptophan standard (**11a**), the presence of 4-, 5-, 6- and 7-bromotryptamine (**7j–7m**) analogs in MIA-CM-10 infers the production of the corresponding bromotryptophans (**11j–11m**; Extended Data Fig. 7b). The demonstration of haloindoles as a viable feedstock for microbial production of halogenated tryptagenic compounds is noteworthy as indole derivatives are substantially cheaper and more accessible than tryptophan or tryptamine derivatives used in previous feeding studies^30^. Furthermore, although *Cro*TDC in MIA-CM-3 was observed to accept all the smaller fluoro and difluoro substitutions, the enzyme showed a strong preference for substitutions at C4 for the larger chloro substitution, and no bromotryptamine (**7j–7m**) could be positively identified (Extended Data Fig. 7c). More promisingly, *Rgn*TDC retained the broad promiscuity reported in vitro, allowing us to report additional promiscuity for 5-, 6- and 7-fluorotryptophan (**7b–7d**) as well as the six different difluorotryptophans (**7n–7s**;^29^ Extended Data Fig. 7d and Supplementary Table 8). Despite this, the extra larger halotryptamines produced by MIA-CM-10 were not generally accepted by *Cro*STR, meaning MIA-CM-3 and MIA-CM-10 each produced seven strictosidine derivatives (**5b–5j**; Extended Data Fig. 7). Based on the broader production of halotryptamines, *Rgn*TDC was selected for further use as the higher general promiscuity was regarded as beneficial for exploring the range of halogenated alstonines possible to produce in a microbial chassis.

**Fig. 3:**
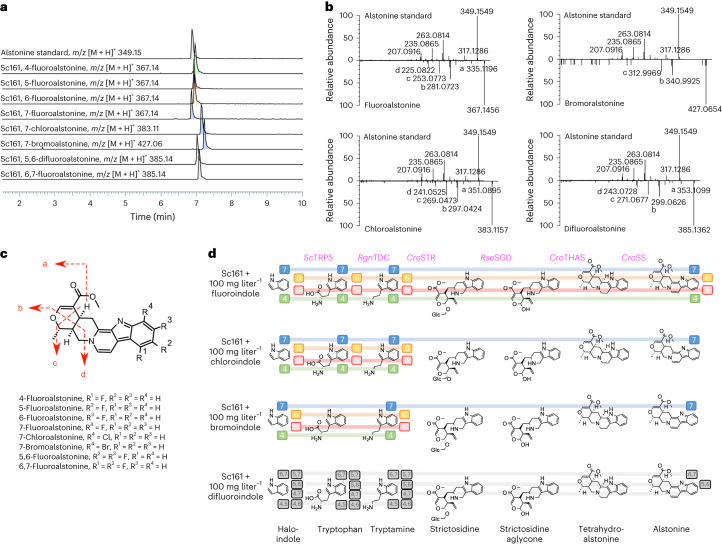
Biosynthesis of halogenated alstonine derivatives in engineered yeast. **a**, Representative liquid chromatography–MS/MS (LC–MS/MS) traces of the chemical standard alstonine and the peaks corresponding to halogenated alstonines after feeding 0.25 mM secologanin and 100 mg liter^–1^ corresponding haloindole derivatives with a single halogen atom at C4 (green), C5 (red), C6 (gold) or C7 (blue). **b**, Representative MS/MS spectra of alstonine standard and fluoroalstonine, chloroalstonine and bromoalstonine. **c**, Peak assignments for alstonine MS/MS spectra shown in **b**. **d**, Progress of the given substitution through the MIA pathway is indicated by the corresponding colored lines. The presence of the colored box indicates direct detection of the halogenated compound after haloindole derivative feeding to cells. Abbreviated enzyme names are stated above the catalyzed reaction. Source data

**Extended Data Fig. 8:**
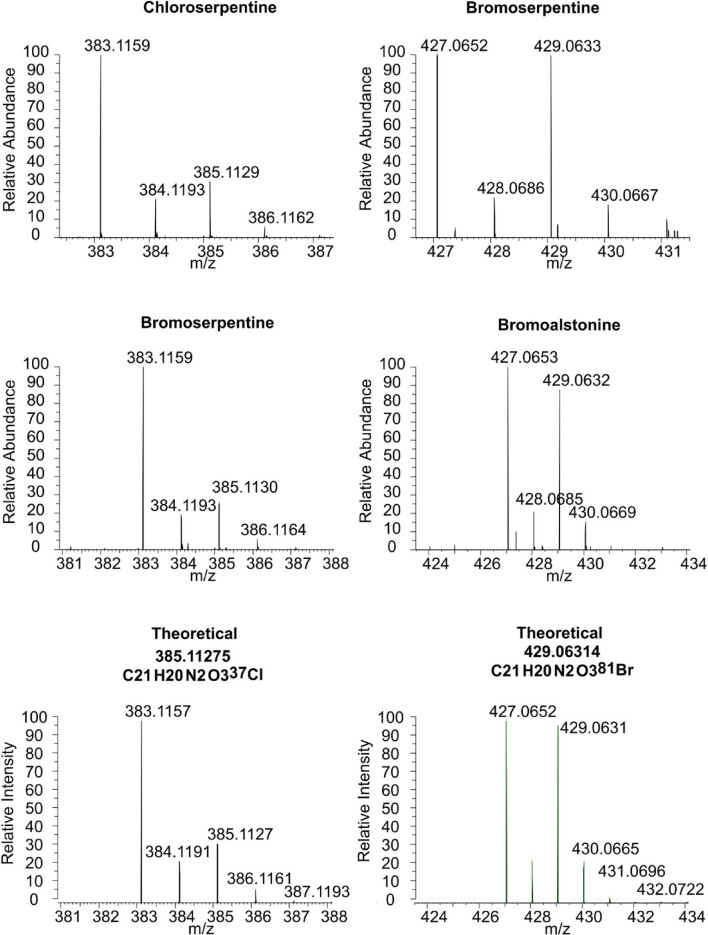
Isotopic profiles of chlorinated and brominated alstonine and serpentine. (Top and Middle panels) Measured isotopic profiles of chlorinated (left) and brominated (right) serpentine and alstonine. (Bottom panels) Predicted isotopic profiles of chlorinated (left) and brominated (right) serpentine and alstonine.

**Extended Data Fig. 9:**
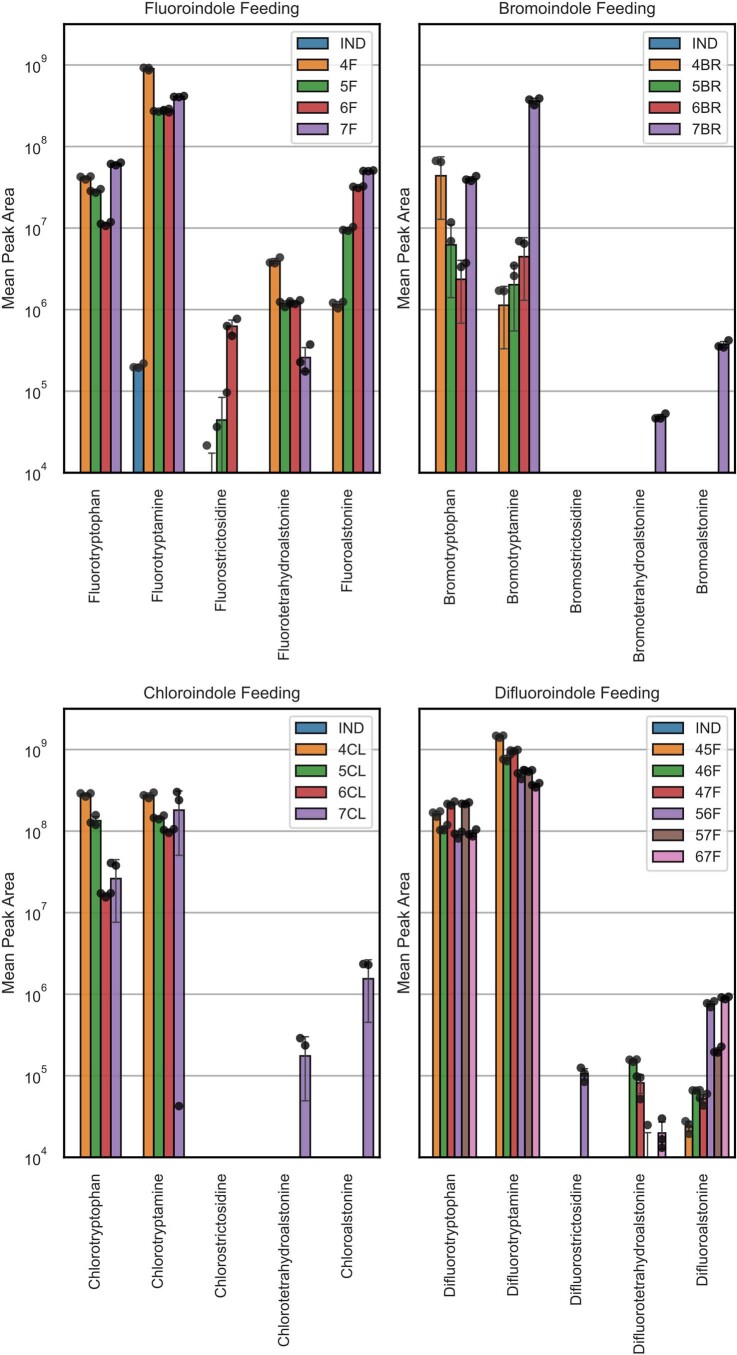
Mean peak areas of halogenated MIA intermediates in Sc161 broth. Sc161 was cultivated in triplicate for 144 h with 100 mg/L halo-indole and 0.25 mM secologanin and the broth analyzed with HRMS. Mean peak areas are plotted with standard deviation and data points overlaid as black dots. Legends indicate the position of halo-substitution(s) according to indole carbon numbering. Source data

Because of the negative growth effects on yeast when supplementing halogenated indoles to the cultivation medium (Supplementary Fig. 7), supplementation was performed following biomass accumulation. Likewise, *Rgn*TDC expression was placed under the control of the galactose-inducible promoter *GAL1* (Sc154) to minimize incorporation of endogenous tryptophan (**11a**) into the MIA pathway before indole feeding. As a further pull on halogenated intermediates toward alstonine analogs, we also introduced *Cro*SS and an additional copy of *Cro*THAS under the control of a galactose-inducible promoter (Sc156). Last, in strain Sc156, we engineered inducible expression of *Cro*SLS (*GAL1* promoter) and overexpression of *INO2* (*TEF1* promoter; Sc159) to mitigate the *Cro*SLS bottleneck and deleted *ROX1* (Sc161) to support P450 enzymes in the pathway^23^. Of these strains, Sc161 produced the most fluoroalstonine (**1b**) in a pilot experiment (Supplementary Fig. 8a) and had the highest signal achieved after 144 h of cultivation (Supplementary Fig. 8b). After feeding the larger 18-membered panel of indole derivatives (**10b–10s**) to Sc161, eight peaks with masses corresponding to 4-, 5-, 6- and 7-fluorinated analogs (**1b–1e**), the 7-chlorinated (**1f**) and 7-brominated analogs (**1g**) and 5,6-difluoroalstonine (**1i**) and 6,7-difluoroalstonine (**1h**) were identified (Fig. 3a). Importantly, all eight alstonine analogs shared the same tandem MS (MS/MS) spectra fragmentation pattern as the alstonine standard and the stereoisomer serpentine^31^(Fig. 3b,c), and the expected isotopic patterns were observed (Extended Data Fig. 8). The detection of all MIA derivatives is summarized in Fig. 3d and Extended Data Fig. 9.

**Extended Data Fig. 10:**
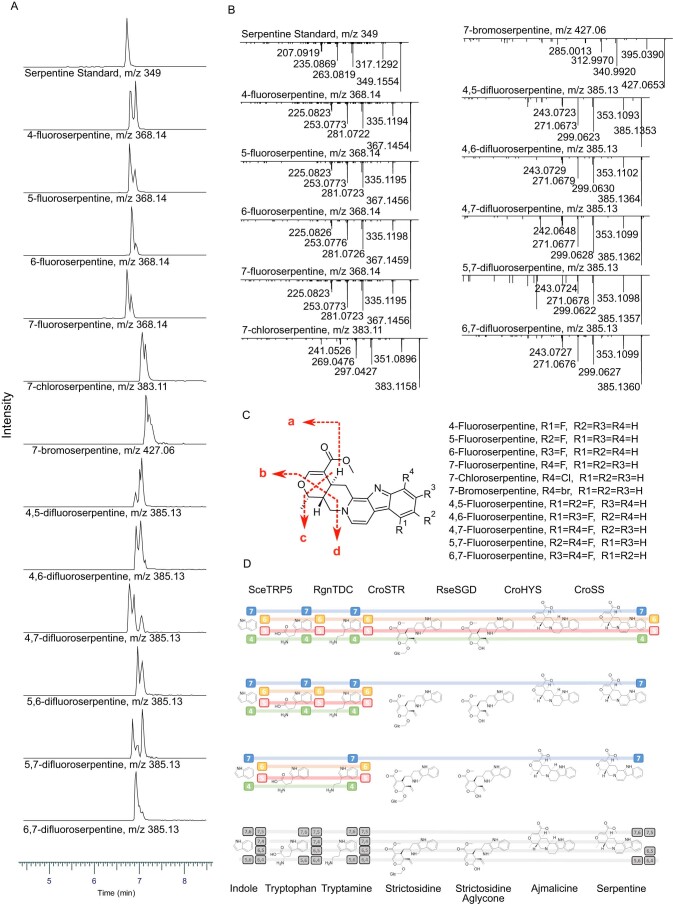
Biosynthesis of halo-serpentines by ScH125. ScH125 was cultivated with 100 mg/L halo-indole and 0.25 mM secologanin. Broth samples were taken at 144 hours and analyzed using HRMS. **a**. Representative chromatograms for serpentine standard and halo-serpentines after feeding the equivalent halo-indole. **b**. Representative MS-MS spectra extracted from the peaks in panel a. **c**. Serpentine and halo-serpentine peak assignment for MS/MS spectra in panel b. **d**. Indole to serpentine pathway expressed in ScH125 with the detection of halogenated derivatives overlaid. The presence of a colored or gray box indicates identification of the derivative with a halo-substitution(s) at the indicated carbon (indole numbering). Enzyme abbreviation: TRP5 - tryptophan synthase 5, TDC - tryptophan synthase, STR - strictosidine synthase, SGD - strictosidine deglycosylase, HYS - heteroyohimbine synthase, SS - serpentine synthase. Source data

Following these findings, cultivations of an Sc154-equivalent serpentine-producing strain expressing *Cro*HYS instead of *Cro*THAS (ScH125; Fig. 1a) resulted in the detection of 11 halogenated serpentine analogs (**2b–2l**; Extended Data Figs. 8 and 10a–d), of which 9 have not been previously reported. The higher number of detected serpentine analogs could be due to *Cro*HYS having either higher overall activity or promiscuity or *Cro*SS having higher promiscuity for ajmalicine derivatives than for THA derivatives. Surprisingly, we observed two distinct peaks in all the halogenated serpentine spectra (Extended Data Fig. 10a). We attribute this to the mixed product profile of *Cro*HYS (Extended Data Fig. 1a), which possibly produces both ajmalicine (**4a**) and THA (**3a**), resulting in both haloalstonine and haloserpentine formation in strain ScH125.

Beyond the detection of novel halogenated MIAs produced in yeast fed with secologanin (**6**) and haloindoles (**10b–10s**), quantitative analysis of MIA pathway intermediates can enable elucidation of biocatalytic bottlenecks. First, a severe bottleneck can be inferred at the Pictet–Spengler reaction catalyzed by *Cro*STR, as, although all 18 tryptamine derivatives (**7b–7s**) are detected, only 12 of these result in a heteroyohimbine analog with a signal strength sufficient for obtaining an MS/MS spectrum (Fig. 3b,d and Extended Data Fig. 9). Of these, the tryptamine derivatives turned over by *Cro*STR are either small (all fluorotryptamines (**7b–7e**) and difluorotryptamines (**7n–7s**)) or positioned on C7 (7-chlorotryptamine (**7f**) and 7-bromotryptamine (**7j**)). The higher promiscuity of *Cro*STR at C7 is in line with previously reported in vitro results^14^, as clashes occur between larger Cl and Br atoms on indole positions C4, C5 and C6 with the *Cro*STR binding site in which the reactive amine moiety is positioned within 2.5 Å of the catalytic glutamate residue^32^.

**Fig. 4:**
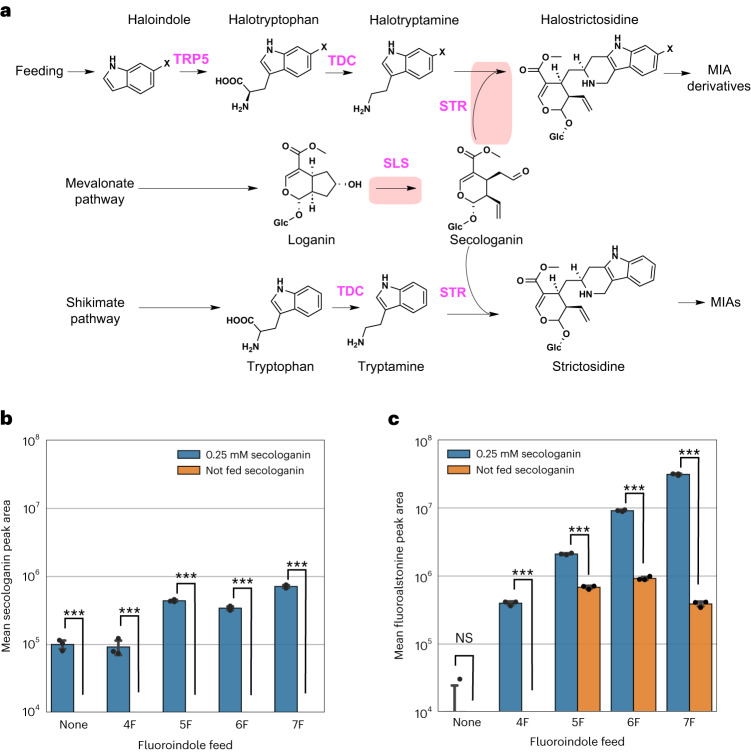
Secologanin is the global limiting substrate and TDC is the limiting enzyme for new-to-nature MIA production in engineered yeast. **a**, Schematic of the natural and derivative MIA pathways competing for the secologanin pool. Bottleneck reactions catalyzed by *Cro*SLS and *Cro*STR are highlighted in red. **b**, Secologanin levels in broth following 144 h of cultivation of Sc154 supplemented with 100 mg liter^–1^ fluoroindole with and without 0.25 mM secologanin. **c**, Fluoroalstonine levels in broth following 144 h of cultivation of Sc154 supplemented with 100 mg liter^–1^ fluoroindole with and without 0.25 mM secologanin. For **b** and **c**, data represent mean values plotted with standard deviation, data points are overlaid as black dots, and statistical significance was calculated using two-tailed Student’s *t*-tests; ***P < 0.001; NS, not significant. Data are shown as means (*n* = 3). Source data

In addition to the steric constraints imposed by *Cro*STR promiscuity, secologanin (**6**) availability also globally restricts flux through this enzymatic step^9^, and competition with natural tryptamine (**7a**) further reduces derivative production (Fig. 4a). Here, unless supplemented at 0.25 mM or heterologously produced by all the strains characterized, secologanin (**6**) was not detectable after 144 h of cultivation (Fig. 4b), and supplementation significantly increased fluoroalstonine (**1b–1e**) signals (Fig. 4c). With respect to the production of alstonine (**1a**) derivatives, when the cultivation medium was supplemented with 0.25 mM secologanin, fluoroalstonine signals also increased significantly (*P* < 10^−4^; Fig. 3b). In summary, secologanin (**6**) availability has a global limiting effect on both natural MIA and derivative production. However, the effect on production of derivatized MIAs is compounded by low *Cro*STR promiscuity, meaning that they are outcompeted by underivatized tryptamine for reaction with secologanin (Fig. 3c).

In summary, this study enabled the microbial production of 8 alstonines (**1b–1i**) and 11 serpentines (**2b–2l**), 9 of which have not been reported before. Additionally, systematic analysis of pathway intermediates demonstrated production of THA (**3b–3i**), ajmalicine (**4b–4l**) and strictosidine aglycone (**20b–20n**) derivatives, while at the same time elucidating limiting metabolic reactions restricting the halogenation of bioactive MIAs.

### De novo biosynthesis of new-to-nature heteroyohimbines

**Fig. 5:**
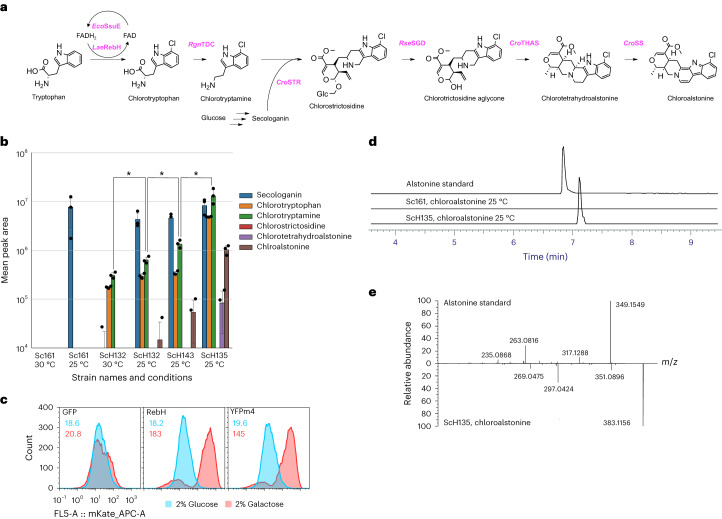
De novo synthesis of chloroalstonine in yeast. **a**, Schematic outline of the de novo chloroalstonine biosynthetic pathway in yeast. **b**, Peak areas for chlorinated MIA masses for halogenase-expressing strains and strain Sc161 (control). Data are shown as mean ± s.d., and data points are overlaid as black dots; *n* = 3. Significance was calculated by using a two-tailed Student’s *t*-test; *P < 0.05. **c**, Detection of *Lae*RebH aggregation with the 4×UAS-SSA1p–365:mKate2 aggregation biosensor. Green fluorescent protein (GFP; left) and yellow fluorescent protein m4 (YFPm4; middle) strains were used as controls for the expression of soluble and aggregation-prone proteins, respectively. Histograms show reporter signals from uninduced (2% glucose) and induced (2% galactose) expression of the candidate proteins. Histograms show all data from *n* = 4 × 4,000 events. Geometric means are reported in the top left. **d**, MS chromatogram traces for alstonine standard and chloroalstonine from ScH125 broth cultured at 25 °C. **e**, Comparison of the MS/MS spectra of an alstonine standard and the chloroalstonine peak in **d** shows a similar fragmentation pattern and a mass shift consistent with a hydrogen-to-chlorine substitution. Source data

To explore the potential of producing new-to-nature MIAs without the need for supplementation of halogenated indoles or secologanin, we next sought to engineer yeast for de novo production of halogenated MIAs. To do so, we integrated a galactose-inducible halogenase expression cassette consisting of the FAD-dependent tryptophan halogenase *Lae*RebH^33^ and the FAD reductase *Eco*SsuE^34^ from *L. aerocolonigenes* and *Escherichia coli*, respectively, into Sc161 (ScH132; Fig. 5a). *Lae*RebH has been shown to catalyze regiospecific bromination and chlorination at C7 of tryptophan and has been previously expressed in *C. roseus*^16^, resulting in the production of halogenated MIAs in planta and the production of halotryptamine in yeast^35^. However, when ScH132 is cultivated at 30 °C with 100 mg liter^–1^ tryptophan (**11a**) and 300 mM NaCl, only chlorotryptophan (**11f**) and chlorotryptamine (**7f**) were observable, yet no downstream chlorinated intermediates were seen (Fig. 5b). As chloroalstonine (**1f**) was previously identified (Fig. 3b,c,d) and natural alstonine (**1a**) was also present in the broth, we reasoned that the limiting factor in chloroalstonine (**1f**) production was chlorotryptamine (**7f**) competition with natural tryptamine (**7a**; Fig. 5b). This hypothesis was addressed by increasing halogenase activity. Functional expression of tryptophan halogenases, such as *Lae*RebH, is notoriously hard to achieve heterologously^36,37^. Indeed, testing a recently developed biosensor for misfolded protein expression in yeast^38^ suggested that a proportion of this enzyme population aggregates in the yeast cytoplasm and is most likely not functional (Fig. 5c and Supplementary Fig. 9). To mitigate this, we repeated the cultivation at a lower temperature (25 °C compared to 30 °C), where secologanin (**6**) production was significantly increased and a peak with an exact mass and retention time consistent with chloroalstonine (**1f**) was detected in the broth from ScH132, albeit with a signal strength too low to obtain a fragmentation pattern required for positive identification (Fig. 5b). However, the chlorotryptamine (**7f**) signal was only mildly improved, even with an extra two copies of the halogenase cassette integrated (Fig. 5b). In a third mitigation approach, we fused a thioredoxin solubility tag to the N terminus of RebH (ScH135)^39^. After cultivating this strain at 25 °C, we observed a greater than tenfold improvement in peak area sufficient to obtain an MS/MS spectrum consistent with that of chloroalstonine (**1f**), resulting in de novo microbial synthesis of a halogenated MIA (Fig. 5b,d,e). To determine if the MIA indole moieties were being directly chlorinated by *Lae*RebH, as opposed to being derived from chlorotryptophan (**11f**), we next fed four different heteroyohimbines (ajmalicine (**4a**), tetrahydroalstonine (**3a**), serpentine (**2a**) or alstonine (**1a**)) to a wild-type strain expressing just the halogenase cassette. In all cases, only chlorotryptophan (**11f**) was observed (Supplementary Fig. 10), meaning that the chlorine entry point in this refactored pathway is indeed tryptophan (Fig. 5a). Last, an attempt to replicate this result with bromoalstonine (**1g**) by cultivating ScH135 with 300 mM KBr resulted in the production of bromotryptophan (**11j**) and bromotryptamine (**7j**), as reported recently^35^, and did not yield any further brominated metabolites (data not shown). The larger size of the bromine atom is likely more inhibitory to flux through the pathway, meaning higher bromotryptamine levels (Fig. 3d) or protein engineering will be required to achieve de novo bromoalstonine biosynthesis.

## Discussion

This work demonstrates a scalable de novo manufacturing platform for bioactive heteroyohimbines in yeast and provides a foundation for further pathway refactoring toward microbial biosynthesis of both native and new-to-nature MIAs^40^. As a proof of principle, we establish biosynthetic pathways for two heteroyohimbines, from which we systematically assess enzyme promiscuity, sterical parameters and metabolic limitations, ultimately enumerating the production of 19 new-to-nature halogenated alstonines and serpentines (Fig. 3d and Extended Data Fig. 10d). Although not all directly detected, this also implies production of at least 12 halogenated strictosidine aglycones (**20b–f**, **20h–n**), 8 halogenated tetrahydroalstonines (**3b–i**) and 11 halogenated ajmalicines (**4b–l**). With >3,000 different MIAs naturally produced in plants and with several plant-sourced MIAs used in the clinic and as traditional medicines in both their natural and derivatized forms^28,41^, the presented platform offers fermentation-based manufacturing and potentially future drug discovery within MIAs, a natural product treasure trove otherwise not easy to source by plant-based extraction or total synthesis.

However, to realize this potential, several challenges need further investigation. First, the inherent promiscuity of enzymes, such as *Cro*HYS and *Cro*SS (Fig. 1 and Extended Data Fig. 10a), can create off-target reactions. This is a particular problem when investigating the ability of natural product pathways to turnover substrate analogs as competing branches can be differentially affected, changing dominant MIA production and complicating efforts to produce specific MIA derivatives in planta^16^. Characterizing the specific product profiles of these enzymes in a yeast cell factory context (for example, using the method described by Yamamoto et al.^12^) and understanding their implications for yields and purification strategies will be important future work. Second, the integration of larger halogens is challenging, as evidenced by the particularly poor passage of the bulky bromine-substituted compounds through the MIA pathway (Fig. 3d and Extended Data Figs. 9 and 10d). Likewise, challenges remain regarding secologanin (**6**) precursor availability and STR promiscuity. Here, further bioprospecting or protein engineering would be warranted^42^.

With this said, enzyme promiscuity can also be an advantage when deploying microbial cell factories as an explorative tool for probing novel regions of chemical space for drug discovery^43^. Although several studies have previously investigated the promiscuity of MIA pathway enzymes for halogenated derivatives in planta^16^, in vitro^14^ and recently in yeast^30,35,44^, this study expands on prior work in the biosynthesis of halogenated MIA scaffolds by (1) systematically assessing the turnover of a wider range of halogenated derivatives by the seven MIA enzymes separating indole from alstonine (**1a**) and serpentine (**2a**; Supplementary Table 8); (2) demonstrating the semisynthesis of 8 derivatives of the bioactive alstonine, including diflourinated MIAs (Fig. 3d), which is notable as successive fluorination has been shown to have additive effects on ligand binding capacity^45^, and 11 serpentine derivatives (9 of which have not been reported before; Extended Data Fig. 10d) and (3) facilitating further study by demonstrating the utility of cheaper indole substrates as a viable derivative entry point into the MIA pathway. In addition to this, the successful biosynthesis of chloroalstonine using *Lae*RebH represents the first de novo production of an MIA derivative in yeast (Fig. 5d,e).

Last, although the fluorinated alstonine (**1b–e**) and serpentine (**2b–e**) analogs are interesting due to overrepresentation in medicinal compounds^46^ and the reported effects on MIA potency^4^, the integration of chlorine and bromine atoms onto the indole moiety may be of greater long-term impact because, as effective leaving groups, they allow specific targeting of the carbon for further derivatization via chemical cross-coupling to organic groups^47^. This would allow the joining of natural and synthetic spheres of chemical space and unlock a near infinite number of MIA derivatives, which could form the future basis of natural product libraries and address the relative lack of drug screening libraries surrounding the privileged indole moiety.

## Methods

### Chemical standards

All chemical standards (Supplementary Table 2) had a purity of 95% or higher.

### Genes

All biosynthetic genes used in the current study are listed in Supplementary Table 3. Genes were synthesized by either Integrated DNA Technologies or Twist Bioscience and optimized for expression in *S. cerevisiae* unless stated otherwise (_nat). The new candidates of AS (*Rte*AS1–*Rte*AS5) were predicted by using the RNA-sequencing reads from project PRJEB39488, downloaded from NCBI. This project contains libraries prepared from young developing leaves and mature and immature leaves and roots. Reads were trimmed with fastp v0.22 (ref. ^48^) and first merged into super reads following the procedure described in the StringTie v2.0 manual using the super-read module from MaSuRCA^49^. Paired reads were then de novo assembled with Trinity v2.8 (ref. ^50^) using the merged super reads as long-read data. Cd-hit was further used to cluster identical and redundant protein sequences^51^. The blast+ suite v2.9 was used to identify putative ASs in the resulting compacted assembly^52^. Alignment and phylogeny were performed in SeaView v4.0 (ref. ^53^).

### Cloning and yeast transformation

All plasmids were constructed by USER cloning^54^ and propagated in *E. coli* DH5α competent cells. Genes for integrative Easy Clone marker-free vectors were prepared according to Jessop-Fabre et al.^55^, and the gRNA expression cassettes were prepared as previously described^56^. All plasmids (Supplementary Table 4) were verified by Sanger sequencing before yeast transformation. The integrative plasmids were linearized by treatment with NotI (New England Biolabs). Yeast transformations were performed using standard lithium acetate methods according to Gietz and Schiestl^57^. The integration of heterologous genes was verified as described in CasEMBLR^55,56^.

### Yeast cultivation and sample preparation

All yeast strains used and constructed in this study (Supplementary Table 5) are based on CEN.PK2-1C, except for the sensing strain used for ADRA2A bioactivity, which was based on BY4741. The de novo strictosidine platform, MIA-CM-3, was recently described by Zhang et al.^9^. To test the yeast strains for production, three colonies were inoculated in 150 μl of SC and incubated overnight at 30 °C and 300 r.p.m. in a 96-well microtiter plate. After 16 h, 10 μl of each culture was transferred into 0.5 ml of fresh medium in a deep-well plate and incubated for 144 h at 30 °C and 400 r.p.m. Yeast strains were cultivated either in YPD or SC. The strains for AS (Sc86–Sc88, Sc90, Sc92, Sc94, Sc96–Sc98, Sc100–Sc104 and Sc157) did not express the strictosidine pathway but only *Cro*STR and downstream genes (Fig. 1a). These strains were fed with 0.25 mM secologanin and 1 mM tryptamine at the start of cultivation. Following 144 h, 100 μl of sample was combined with 100 μl of caffeine (900 μg liter^–1^) and filtered through a filter plate (PALL, AcroPrep Advance, 0.2-μm Supor membrane for medium/water) by centrifugation at 2,200*g* for 1 min. Samples (2 μl) samples were injected for analytical measurements.

For the production of halogenated MIAs using haloindole feeding, triplicate overnight cultures in 18 ml of 3× SC and 2% glucose supplemented with 20 g liter^–1^ bovine peptone (Sigma-Aldrich) were incubated for 72 h at 30 °C or 25 °C and 300 r.p.m. in a baffled shake flask. Each culture was centrifuged at 3,000 r.p.m. for 5 min, the supernatant was discarded, and the pellet was washed three times in 10 ml of PBS with 5% ethanol and resuspended in 6 ml of 3× synthetic defined (SD) medium without tryptophan supplemented with 2% galactose and 0.25 mM secologanin. The optical density at 600 nm (OD_600_) was then corrected to 80. The cell suspension (270 μl) was transferred to a 96-well deep-well plate, and 30 μl of 1 g liter^–1^ indole, 4-fluoroindole, 5-fluoroindole, 6-fluoroindole, 7-fluoroindole, 4-chloroindole, 5-chloroindole, 6-chloroindole, 7-chloroindole, 4-bromoindole, 5-bromoindole, 6-bromoindole, 7-bromoindole, 4,5-difluoroindole, 4,6-difluoroindole, 4,7-difluoroindole, 5,6-difluoroindole, 5,7-difluoroindole or 6,7-difluoroindole dissolved in 3× SC plus 5% acetone was added to the well. The plate was incubated for 144 h at 30 °C or 25 °C and 400 r.p.m. After 72 h, 15 µl of 2% galactose was spiked into each well. After 144 h, 100 μl of sample was filtered through a filter plate (PALL, AcroPrep Advance, 0.2-μm Supor membrane for medium/water) by centrifugation at 2,200*g* for 1 min. Samples (1 μl) were injected for analytical measurements. An identical protocol was followed for de novo integration of halogens, except 30 μl of 3× SD supplemented with 1 g liter^–1^ tryptophan and 3 M NaCl or KBr was added instead of 30 μl of indole.

### Analytical methods

For routine sample analysis, an advance UHPLC system (Bruker Daltonics) coupled to an EVOQ Elite triple quadrupole mass spectrometer (Bruker Daltonics) was used. The applied column was a 100-mm C18 Acquity UPLC HSS T3 column (100 Å) with a 1.8-μm particle size and 2.1-mm inner diameter (Waters); with [KBA1], the column oven temperature was 35 °C. The mobile phase consisted of Milli-Q water (solvent A) and acetonitrile (solvent B) both with 0.1% formic acid. The flow rate was 0.5 ml min^–1^ with the following gradient profile: isocratic 0–0.8 min at 5% B, gradient 0.8–4 min from 5 to 55% B, gradient 4–4.2 min from 55 to 95% B and isocratic 4.2–5.5 min at 95% B. The column was reequilibrated for ~2 min before the next injection, and the instrument was navigated by MS Workstation software version 8.210.0.253 (Bruker Daltonics). Multiple reaction monitoring data (Supplementary Table 6) were collected by electrospray ionization that operated in positive ion mode with the following parameters: spray voltage of 4.5 kV, cone temperature of 350 °C, cone gas flow of 20, probe gas flow of 50, nebulizer gas flow of 50, heated probe temperature of 300 °C, exhaust gas on and a collision-induced dissociation of 1.5 mTorr. Analyte stock solutions were prepared in ethanol for tryptamine, tetrahydroalstonine, ajmalicine, serpentine and alstonine and in Milli-Q water for loganic acid, loganin, secologanin and strictosidine. Ten levels of calibration were prepared, diluting the standard mixture with SC or YPD (Supplementary Table 6). The calibration curves for alstonine and ajmalicine were prepared separately to avoid their interferences on coeluting isomers serpentine and THA, respectively. Caffeine (0.9 mg liter^–1^) was used as an internal standard in standards and samples. The calibration levels were weighted as 1/*x*, and the curve was fitted by linear regression. When the concentration of MIAs in the samples exceeded the highest concentration used in the calibration curve, the samples were diluted.

To confirm the reported activity of *Cro*THAS1 and *Cro*HYS, when heterologously expressed in yeast, method 2 described by Stavrinides et al.^20^ was applied. To confirm the identity of alstonine and serpentine, which has the same retention time in the routine LC–MS method applied in this study, we compared LC–MS transition profiles to the ones obtained by high-resolution MS (LC–HRMS). Both standards along with the alstonine de novo producers were included. When the two stereoisomers were compared by both methods, the ratio of the fragment ion 316.9 was comparatively higher for serpentine than alstonine (Extended Data Fig. 6). The analysis of halogenated MIAs was performed on a Vanquish Duo UHPLC binary system (Thermo Fisher Scientific) coupled to an Orbitrap ID-X mass spectrometer (Thermo Fisher Scientific). The data were acquired in positive mode with a voltage of 3,500 V, and the rest of the parameters were as described previously by Kildegaard et al.^58^.

For detection of halogenated compounds, LC–MS/MS analysis was performed using a Vanquish Duo UHPLC binary system (Thermo Fisher Scientific) coupled to an Orbitrap ID-X Tribrid mass spectrometer (Thermo Fisher Scientific). Chromatographic separation was achieved under reverse-phase conditions as previously described^58^. The MS measurements were performed in positive-heated electrospray ionization mode with a voltage of 3,500 V acquiring the full MS/MS spectra (data-dependent acquisition-driven MS/MS) in the mass range of 70–100 Da. The following data-dependent acquisition settings were used: automatic gain control target value of 4 × 10^5^ for full-scan MS and 5 × 10^4^ for the MS/MS spectral acquisition and a mass resolution of 120,000 for full-scan MS and 30,000 for MS/MS events. Precursor ions were fragmented by stepped high-energy collision dissociation using collision energies of 20, 40 and 60. As a general note, as standards for halogenated MIA analogs are not commercially available, halogenated MIAs were identified based on exact mass and retention times and MS/MS spectra shifts relative to the unhalogenated standard, as previously described^30,45,59^ (Supplementary Table 9).

### Glucose, ethanol and organic acids

Glucose, ethanol and organic acids were analyzed using a Rezex ROA-Organic Acid H^+^ (8%; 150 × 7.8 mm) column on a Vanquish dual-system HPLC coupled with a refractive index (IDEX RefractoMax 521) and diode array detector. The HPLC was run with a flow rate of 0.7 ml min^–1^ (isocratic) using 9 mM sulfuric acid at a column temperature of 60 °C. The injection loop volume was kept to 5 µl for all samples and standards. For diode array detection, a 210-nm wavelength was used for detection and confirmation of organic acids. Peak identification was based on the relative retention times determined by injection of standard solutions. Quantification of compounds was performed using calibration curves on Chromeleon software, version 7.2.8.

### Microbioreactor

For fed-batch cultivations, the microbioreactor screening platform BioLector Pro coupled to RoboLector (mp2-labs) was used. The microbial cultures were cultivated in microfluidic flower plates (MTP-MF32C-BOH2, mp2-labs) in triplicate with a starting OD_600_ of 0.5 and 1-ml volume, essentially as described previously^9^. The process started with 20 h of batch phase (3× SC, 2% glucose and 3 mM tryptophan) followed by exponential feeding with 36% glucose at a 0.48*exp(0.0125*t*) feeding rate until 144 h. To test the effect of supplementation of the medium on alstonine production, 10 g liter^–1^ soy peptone (Sigma-Aldrich) or 10 g liter^–1^ bovine peptone (Sigma-Aldrich) was added to the medium, the culture was maintained at 30 °C and 1,000 r.p.m., and the pH was maintained at 5.5 with the automatic addition of 10% NH_4_OH solution. The relative humidity in the growth chamber was maintained at 85% to minimize evaporation of the medium. The biomass, pH and dissolved oxygen were recorded during the run using built-in optical sensors. The cultures were sampled at 48, 96 and 144 h, processed as described in Yeast cultivation and sample preparation and analyzed for glucose, ethanol, organic acids and MIAs (routine LC–MS method). Biomass was determined using 0.5 ml of each sample dried at 60 °C overnight.

### Microtiter plate reader

Triplicate overnight cultures of 1 ml of SC plus 2% glucose were inoculated in 15-ml cultivation tubes and incubated overnight at 30 °C. One hundred and fifty microliters of each overnight culture was diluted 100-fold in SC plus 2% glucose. Then, 135 µl of the 100-fold-diluted cell suspension was added to a clear-bottomed 96-well plate. Fifteen microliters of 1 g liter^–1^ haloindole in 3× SD and 20% DMSO was added to each well. The plate was then covered with a Breathe-Easy sealing membrane (Diversified Biotech BEM-1) and incubated in a SynergyMX microtiter plate reader (BioTek) at 30 °C and 250 r.p.m., and the OD_600_ was measured every 20 min for 48 h. Growth curves were processed and analyzed using a Python regression analysis script^60^.

### Two-liter bioreactor procedures for Sc112 and FH144

Fed-batch cultivations at 2-liter scales were performed and monitored as previously described^61^ with the pH set to 5.5. The process started with 20 h of batch phase (3× SC, 2% glucose and 3 mM tryptophan), followed by a fed-batch phase. Sc112 was fed as described for the microbioreactor experiments with a 6% glucose feed. ScH144 was fed with pulses of the batch medium concentrated ten times without tryptophan with 200 g liter^–1^ glucose or 153.5 g liter^–1^ ethanol (equivalence of carbon molarity). For pulsed fed-batch, yeast nitrogen base concentration in the feed was decreased by three after 95 h of feeding to avoid over osmotic pressure.

### Purification of alstonine

Purification of alstonine for NMR and bioactivity tests included a four-step procedure based on liquid–liquid extraction, silica pretreatment and two repeated steps of purification by LC. First, for liquid–liquid extraction, cultivation broth from controlled bioreactors was collected based on centrifugation and collection of 1.5 liters of supernatant. Next, two rounds of liquid–liquid extraction with 1:1 dichloromethane:broth were performed, and the aqueous phase was concentrated by vacuum. For silica pretreatment, the dried extract was solubilized with water and methanol and then a normal phase of silica (Fuji Silysia, Chromatorex GS60 20/45 µm). Following subsequent homogenization, this solution was concentrated under vacuum to give a dry extract, and the dried extract was purified using silica gel (Fuji Silysia, Chromatorex GS60 20/45 µm) column chromatography with a different ratio of methylene chloride:methanol (100:0–0:100), yielding the first crude alstonine extract. Fractions containing alstonine were then pooled and concentrated to dryness, from which the amount of product was approximately 4 g with a purity of alstonine at 250 nm of <1%. Following this, the first LC step of purification was performed with a Dynamic Axial Column with a 50-mm diameter. The silica gel used was Kromasil C18 5 µm (300 g), with a mobile phase consisting of water with 0.1% formic acid:acetonitrile (80:20 (vol/vol)) and washing solvent consisting of 70:30 (vol/vol) formic acid:acetonitrile and a flow rate of 70 ml min^–1^. For diode array-based detection, a 250-nm wavelength was used for detection. All fractions containing alstonine were pooled, dry distilled and concentrated under vacuum. From this, 100 mg of alstonine was recovered with 10% relative purity by HPLC. Last, a second step of purification was performed on a semipreparative system. Here, the silica gel used was Aquasil C18 5 µm (250 × 20 mm), with a mobile phase consisting of water with 0.1% formic acid:methanol (65:35 (vol:vol)) and washing solvent consisting of 20:80 (vol:vol) formic acid:acetonitrile. The flow rate was fixed at 8 ml min^–1^. For diode array-based detection, a 250-nm wavelength was used. For the second purification step, the quantity injected was fixed at 50 mg per run, and all fractions containing alstonine were again pooled, dry distilled and concentrated under vacuum. From this, we obtained 2 mg of alstonine, which was recovered with 95% relative purity by HPLC.

### SPE purification

Before NMR, the alstonine sample was purified by short-path chromatography using an SPE cartridge (Discovery DPA-6S, 250 mg, volume 3 ml (Supelco)). The SPE cartridge was conditioned with 3 column volumes of deionized water and equilibrated with 5 column volumes of methanol. The product was loaded with a small amount of methanol and eluted by methanol. The eluted solution was collected as fractions, and each fraction was checked by LC–MS. The purest fraction was dried under N_2_ stream and subjected to NMR measurement.

### NMR methods

NMR measurements were performed on a 700 MHz Bruker Avance III HD spectrometer (Bruker Biospin) equipped with a TCI cryoprobe using standard pulse sequences, as implemented in Bruker Topspin version 3.6.1 (Bruker Biospin). Chemical shifts were referenced to the residual solvent signals of methanol-*d*_*3*_ (*δ*_H_ 3.31).

### NMR data of alstonine from fermentation as formic acid salt

^1^H NMR (700 MHz, methanol-*d*_*3*_): *δ* ppm: 1.48 (d, *J* = 6.2 Hz, 3H), 2.56 (dddd, *J* = 9.7, 5.8, 5.6, 5.1 Hz, 1H), 3.21 (dddd, *J* = 9.3, 6.3, 6.2, 5.6 Hz, 1H), 3.37 (dd, *J* = 17.5, 9.3 Hz, 1H), 3.79 (s, 3H), 4.02 (dq, *J* = 9.7, 6.2 Hz, 1H), 4.17 (dd, *J* = 17.5, 6.3 Hz, 1H), 4.69 (dd, *J* = 14.3, 5.1 Hz, 1H), 5.03 (dd, *J* = 14.3, 5.8 Hz, 1H), 7.46 (dd, *J* = 8.0, 7.5 Hz, 1H), 7.73 (s, 1H), 7.75 (d, *J* = 8.2, 1H), 7.79 (dd, *J* = 8.2, 7.5 Hz, 1H), 8.38 (d, *J* = 8.0, 1H), 8.46 (d, *J* = 6.4, 1H) and 8.52 (d, *J* = 6.4, 1H). The chemical shifts were in agreement with published data^62^.

### Assessment of RebH aggregation

Protein aggregation was assessed in strains expressing three copies of target proteins expressed from Gal1p integrated in LP3 landing pads^63^ and the aggregation reporter 4×UAS-SSA1p–365:mKate2 (ref. ^38^). A strain expressing GFP was used as a non-aggregation control, and a strain expressing the non-fluorescent and aggregation-prone target YFPm4 (ref. ^64^) was used as a positive control for aggregation. Strains expressing YFPm4 and RebH also expressed the Hsp104–GFP fusion protein to visualize aggregates. Assessment of aggregates was done in cultures of single colonies in 96-well plates with four replicates per condition, totaling three strains (GFP, YFPm4 and RebH strains) and two conditions (2% glucose and 2% galactose media). Precultures were grown overnight in 150 µl of SC medium with 2% glucose at 30 °C and 300 r.p.m. Cultures were diluted 75-fold in SC with 2% glucose or 2% galactose and incubated for 16 h at 30 °C at 300 r.p.m. Samples were taken for flow cytometry (reporter 4×UAS-SSA1p–365:mKate2) and microscopy. For assessment of aggregation using the 4×UAS-SSA1p–365:mKate2 aggregation reporter, samples of strains expressing target proteins were assessed in a MACSQuant Analyzer VYB flow cytometer (Miltenyi Biotec), totaling four distinct samples per condition. Cell populations were gated for singlets using an FSC-A versus FSC-H plot, and 4,000 singlet events were recorded per sample. A 561-nm laser and 661/20-nm filter were used for the mKate2 measurements. For observation of protein aggregates by microscopy, strains were grown overnight in 96-well plates in 150 µl of SC medium with 2% glucose at 30 °C and 300 r.p.m. Cultures were diluted 1:75 in SC with 2% galactose for 16 h at 30 °C and 250 r.p.m. Aggregates were observed with a Leica DM4000 B microscope equipped with a DFC 300 FX R2 camera and an EL600 light source (Leica Microsystems). Excitation/emission filters used for GFP were 470/40 nm and 525/50 nm, respectively.

### Alstonine bioactivity assays

Yeast strain Sc272 expresses NanoLuc after stimulation of the ADRA2A receptor. Sc272 was generated by changing the superfolder GFP reporter to NanoLuc and integrating ADRA2A in the base strain yWS2267 (ref. ^65^). This design was inspired by the similar ADRA2A-sensing yeast described in ref. ^26^, and the luciferase assay was performed essentially as previously reported^66^. Before the bioactivity assay, Sc272 was inoculated from cryostock into 5 ml of SC-tryptophan and incubated overnight at 30 °C and 250 r.p.m. Next, 2 ml of culture was centrifuged at 5,000*g* for 5 min, and the supernatant was discarded. The pellet was then resuspended in pH-buffered SC-tryptophan at pH 7.2. The OD_600_ was measured and adjusted to 1.25 before 40 µl was distributed to sterile 96-well plates (Greiner, 655101) and covered with a Breathe-Easy sealing membrane (Diversified Biotech BEM-1). The plates were incubated for 2 h at 30 °C and 250 r.p.m. before the membrane was removed. Then, 5 µl of 12 different 10× stock concentrations of epinephrine, yohimbine or alstonine containing 10% (vol/vol) DMSO was added in triplicate series. Milli-Q water (5 µl) was then added to the wells with epinephrine, whereas 5 µl of 500 µM epinephrine in Milli-Q water was added to wells with alstonine or yohimbine. The plates were covered again and incubated for 4 h at 30 °C and 250 r.p.m. Twenty minutes before incubation was completed, a lysis mix of 1.33% (vol/vol) furimazine (NanoLuc substrate) from Promega in CelLytic Y cell lysis reagent (Sigma-Aldrich) was mixed, and 15 µl was distributed to wells of a white, small-volume 96-well plate (Greiner, 675083). Following incubation, the plate was vortexed for 5 s before the membrane was removed, 5 µl of each well containing sensing cells was transferred to the plate containing the lysis mix, and the plate was immediately placed in a SynergyMX microtiter plate reader (BioTek). Luminescence was measured at 12, 13.5 and 15 min after mixing. The settings were filter luminescence, with a gain of 110 and 0.5-s integration per well; the average of each of the time points is reported as single replicates per well.

For the bioactivity assay of alstonine on the 5-HT_2C_ receptor conducted in COS7 cells, the level of cAMP was monitored using bioluminescence resonance energy transfer (BRET). This method is based on a construct consisting of a cAMP-binding protein (exchange protein activated by cAMP (Epac)), which is flanked by a BRET pair (*Renilla* luciferase (Rluc) and YFP). Together, this complex is called cAMP sensor using YFP–Epac–Rluc (CAMYEL)^67^. cAMP production is sensed as Epac changes conformation in response to the increasing levels of cAMP, leading to a loss of BRET intensity. COS7 cells were plated in poly-d-lysine-coated, white 96-well plates (20,000 cells per well). The following day, cells were transfected in 100 μl of transfection medium per well consisting of 20 µg of plasmid pFH78 and 100 µg of CAMYEL per 10 ml of medium for a total of 5 h and thereafter incubated in 100 μl of growth medium overnight. The next day, cells were washed twice with 100 μl of HBSS per well (Gibco, Life Technologies) and preincubated for 30 min at 37 °C with 60 μl of HBSS. The luciferase substrate coelenterazine (Thermo Fisher Scientific) was added, and, after a 5-min incubation, a baseline was measured. Ligands were added, and measurements were recorded every minute for 30 min using a CLARIOstar Plus plate reader. The BRET signal was calculated as the ratio of the emission intensity at 535 nm (citrine) to the emission intensity at 475 nm (luciferase). Determinations were made in triplicate. Dose–response curves were generated using the non-linear regression function in GraphPad Prism version 9.5.0 for Windows from GraphPad Software. For the ADRA2A receptor, the model used the variable slope setting, whereas the model for 5-HT_2C_ assumed Hill coefficients of 1 and −1 for stimulation and inhibition curves, respectively.

### Reporting summary

Further information on research design is available in the Nature Portfolio Reporting Summary linked to this article.

## Online content

Any methods, additional references, Nature Portfolio reporting summaries, source data, extended data, supplementary information, acknowledgements, peer review information; details of author contributions and competing interests; and statements of data and code availability are available at 10.1038/s41589-023-01430-2.

### Supplementary information

Supplementary Information Supplementary Figs. 1–10 and Tables 1–9.

Reporting Summary

Supplementary Data Excel file containing source data for the Supplementary figures.

### Source data

Source Data Fig. 1 Statistical source data.

Source Data Fig. 2 Statistical source data.

Source Data Fig. 3 Statistical source data.

Source Data Fig. 4 Statistical source data.

Source Data Fig. 5 Statistical source data.

Source Data Extended Data Fig. 1 Statistical source data.

Source Data Extended Data Fig. 2 Statistical source data.

Source Data Extended Data Fig. 3 Statistical source data.

Source Data Extended Data Fig. 6 Statistical source data.

Source Data Extended Data Fig. 7 Statistical source data.

Source Data Extended Data Fig. 9 Statistical source data.

Source Data Extended Data Fig. 10 Statistical source data.

## Data Availability

Data supporting the findings of this work are available within the article, Extended Data figures and Supplementary Information. The datasets generated and analyzed during the current study are also available from the corresponding authors upon request. Source data are provided with this paper.
