# Supplementary material for: Biosynthesis of natural and halogenated plant monoterpene indole alkaloids in yeast
Source: Nat Chem Biol. 2023 Nov 6;19(12):1551–60. doi: 10.1038/s41589-023-01430-2 (PMC10667104; doi:10.1038/s41589-023-01430-2)
Supplement: Supplementary file 1 — Supplementary Figs. 1–10 and Tables 1–9. [file 41589_2023_1430_MOESM1_ESM.pdf]

nature chemical biology

Article

https://doi.org/10.1038/s41589-023-01430-2

Biosynthesis of natural and halogenated plant monoterpene indole alkaloids in yeast

In the format provided by the authors and unedited

Contents of Supplementary Information

Supplementary Figure 1. Levels of pathway intermediates from the best alstonine and serpentine *de novo* producing strains, cultivated in deep-well plates.

Supplementary Figure 2. Production of alstonine, serpentine and pathway intermediates by two additional replicates of strains Sc112 and Sc85 when using the fed-batch process.

Supplementary Figure 3. Levels of tryptamine accumulated in the strains cultivated in the fed-batch process at 48 h, 96 h and 144 h.

Supplementary Figure 4. Levels of glucose, ethanol and acetic acid in strains cultivated in fed-batch process.

Supplementary Figure 5. Production of alstonine in yeast.

Supplementary Figure 6. Main extracellular metabolite levels and biomass during fed-batch fermentations at 2 liter scale.

Supplementary Figure 7. Growth effect of halo-indoles on yeast.

Supplementary Figure 8. Fluoro-alstonine production in strain Sc156, Sc159 and Sc161.

Supplementary Figure 9. Microscope images of *Lae*RebH expressing strains show aggregation in yeast.

Supplementary Figure 10. *Lae*RebH is specific to tryptophan.

Supplementary Table. 1. Screen of alstonine synthase candidates in SC and yeast-extract/peptone/dextrose (YPD) cultivation medium.

Supplementary Table. 2. Chemical standards used in this study.

Supplementary Table 3. Genes in this study.

Supplementary Table 4. Plasmids used and constructed in this study.

Supplementary Table 5. Strains used and constructed in this study.

Supplementary Table 6. Metabolite retention times (RTs) and quantifier and qualifier fragments used for Multiple Reaction Monitoring (MRM).

Supplementary Table 7. Concentration of compounds used in analytical standards.

Supplementary Table 8. Prior art and results from this study on promiscuity of MIA enzymes for halogenated substrate derivatives.

Supplementary Table 9. Metabolite retention times, masses and identifying fragments used in HRMS.

Supplementary Figures

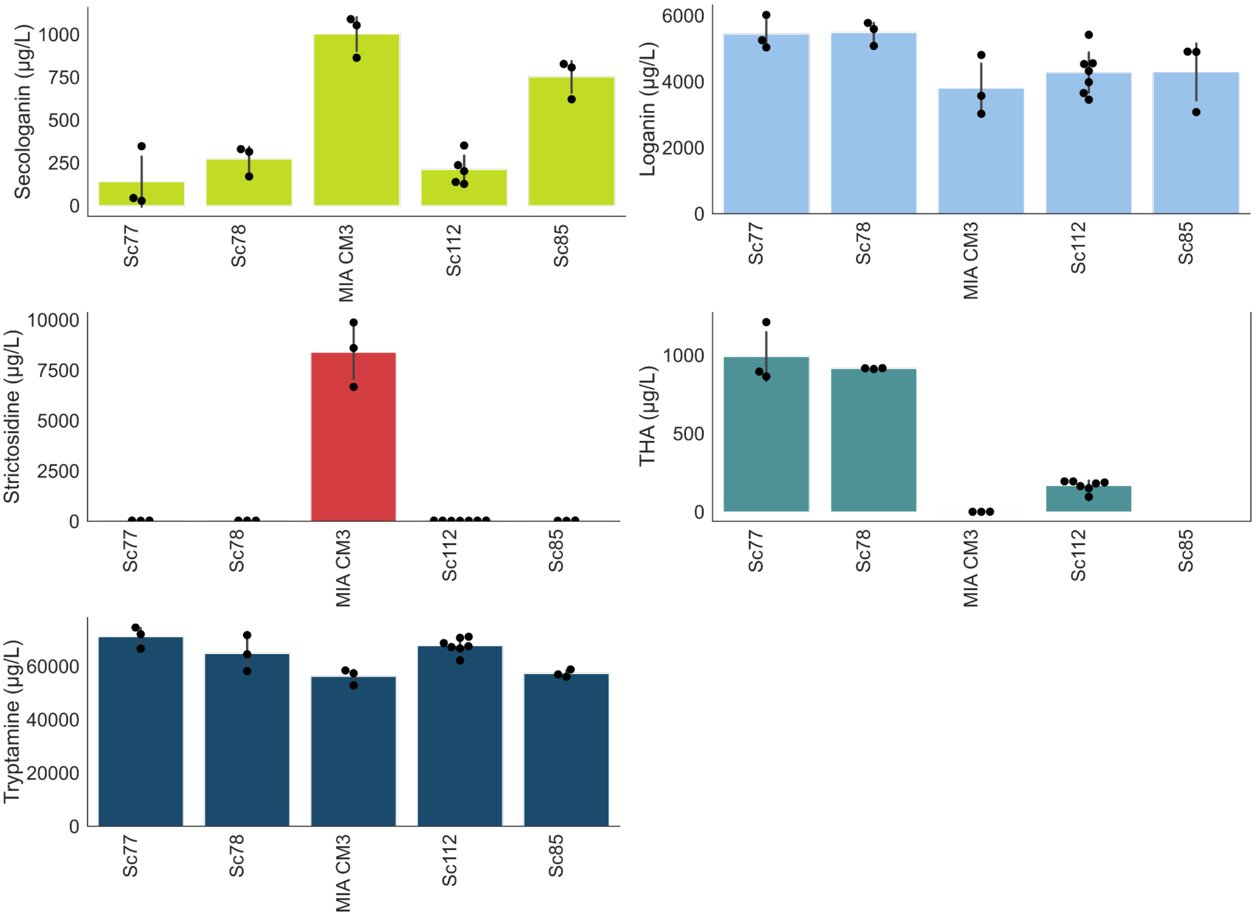

Supplementary Figure 1. Levels of pathway intermediates from the best alstonine and serpentine *de novo* producing strains, cultivated in deep-well plates. Pathway intermediates (µg/L) produced by Sc77, Sc78, MIA-CM-3 (Control, [1]), and Sc112, when cultivated in 3 x SC + 3mM Trp medium. The strains Sc77 and Sc78 are expressing *Cro*THAS1+*Rte*AS2 and *Cro*THAS1+*Gse*SBE_nat, respectively, whereas strain Sc112 is expressing *Cro*THAS1+*Cro*SS and strain Sc85 is expressing *Cro*HYS+*Cro*SS. All the strains are based on MIA-CM-3, with a genome integrated copy of *Rse*SGD. Secologanin (light green), tryptamine (Dark blue), THA (olive), strictosidine (red), loganin (light blue). THA in Sc85 was not quantified due to the presence of ajmalicine. Mean, n=3 (except Sc112 with n=6), with error bars indicating SD.

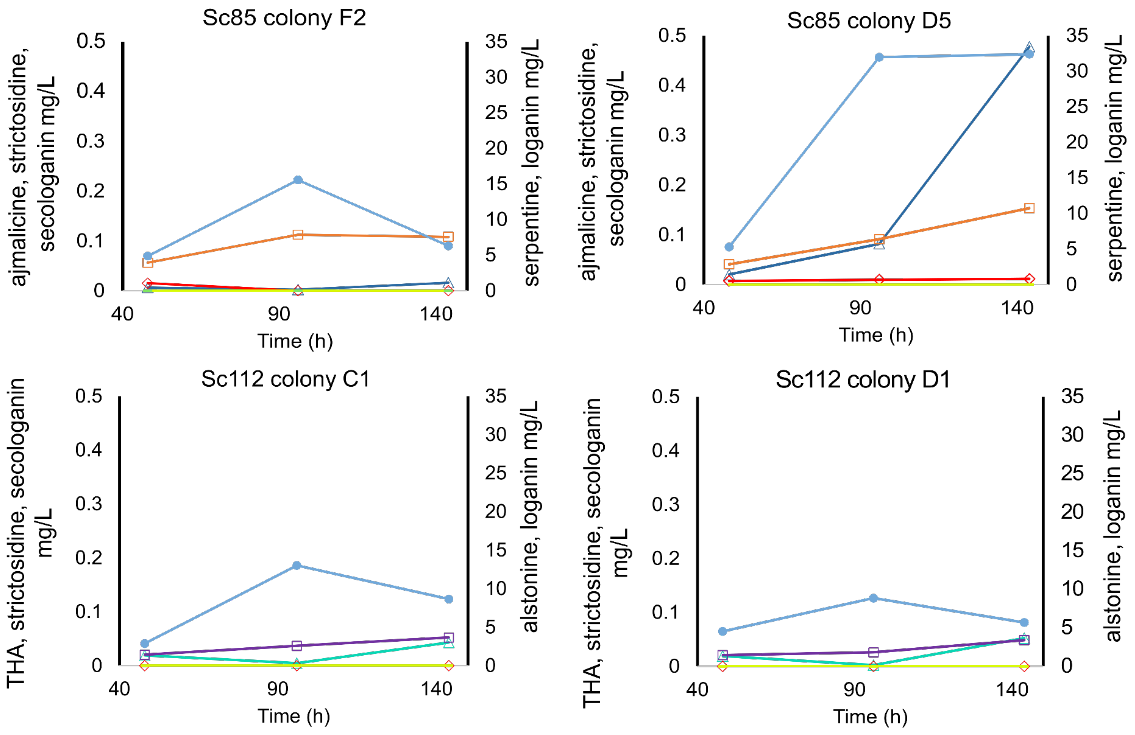

Supplementary Figure 2. Production of alstonine, serpentine and pathway intermediates by two additional replicates of strains Sc112 and Sc85 when using the fed-batch process. The Biolector cultivated strains were growing in 3 x SC medium supplemented with 3 mM Trp and fed with glucose after 20h for additional 124 h. The strain Sc85 (colony F2 and D5) is expressing *Cro*HYS, *Rse*SGD and *Cro*SS. Serpentine (orange, □), loganin (blue, ●), ajmalicine (dark blue, Δ), strictosidine (red, (◊), secologanin (light green, -). The strain Sc112 (colony C1 and D1) is expressing *Cro*THAS1, *Rse*SGD and *Cro*SS. Alstonine (purple, □ 6), loganin (blue, ● 5), tetrahydroalstonine (THA)(cyan, Δ 7), strictosidine (red, ◊ 8), secologanin (light green, - 5). Both strains are based on MIA-CM-3, the *de novo* strictosidine strain. n=2 individual graphs

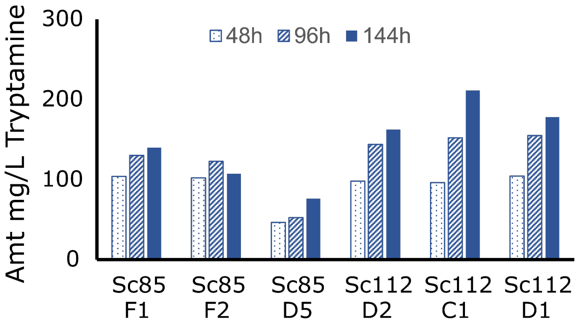

Supplementary Figure 3. Levels of tryptamine accumulated in the strains cultivated in the fed-batch process at 48 h, 96 h and 144 h. The Biolector-cultivated strains were grown in 3 x SC medium supplemented with 3 mM Trp, and fed with glucose after 20 h for an additional 124 h at exponential feeding rate (0.48*exp(0.0125t. The strain Sc85 (colonies F1, F2 and D5) is expressing *Cro*HYS, *Rse*SGD and *Cro*SS, and the Sc112 (colonies D2, C1 and D1) is expressing *Cro*THAS1, *Rse*SGD and *Cro*SS. All the strains are based on MIA-CM-3 [1]**.** n = 3, individual bars.

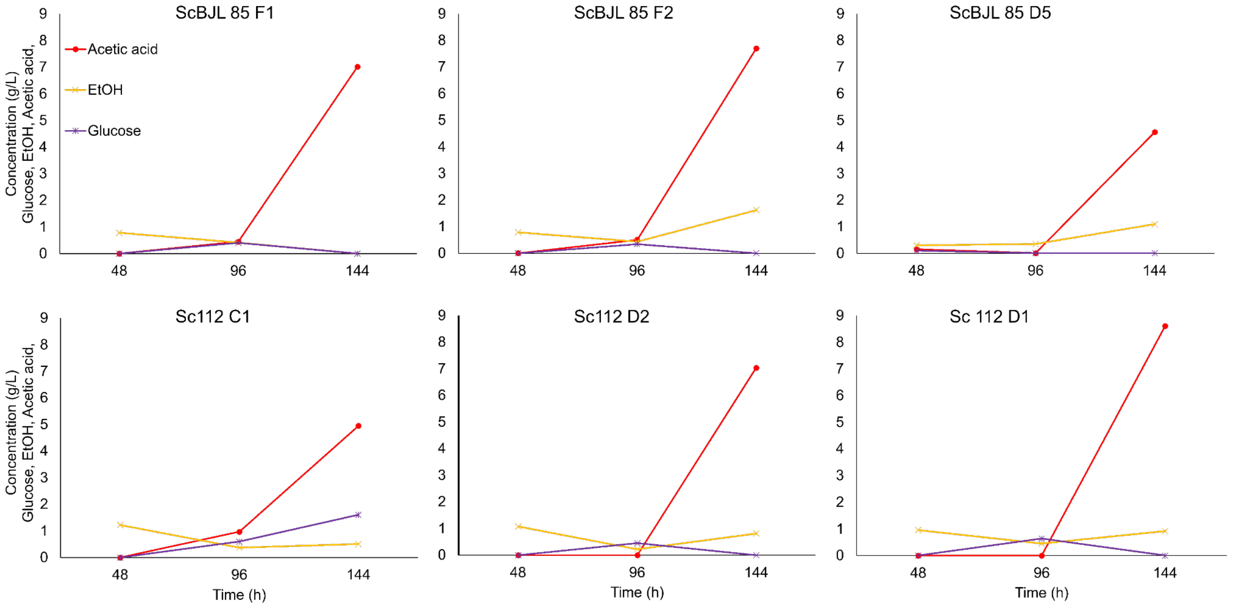

Supplementary Figure 4. Levels of glucose, ethanol and acetic acid in strains cultivated in fed-batch process. The Biolector cultivated strains were growing in 3 x SC medium supplemented with 3 mM Trp and fed with glucose after 20 h for additional 124 h at exponential feeding rate (0.48*exp(0.0125t). Levels of glucose (purple *), ethanol (yellow x) and acetic acid (red ●) in the microbioreactor cultures.The strain Sc85 (colony F1, F2 and D5) is expressing *Cro*HYS, RseSGD and *Cro*SS. And the Sc112 (colony D2, C1 and D1) is expressing *Cro*THAS1, *Rse*SGD and *Cro*SS. All the strains are based on MIA-CM-3, the *de novo* strictosidine strain [1]. n = 3, individual graphs.

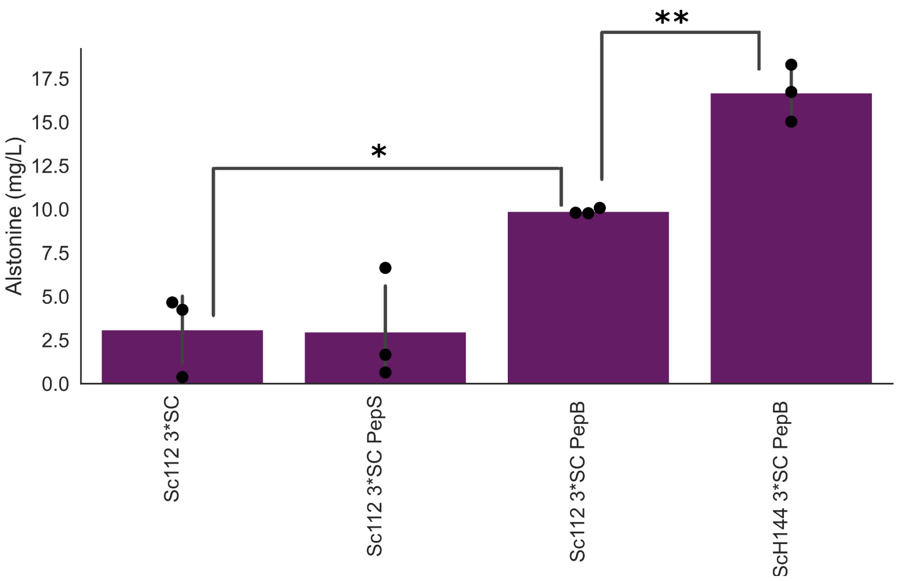

Supplementary Figure 5. Production of alstonine in yeast. Strains Sc112 and ScH144 cultivated in microbioreactor for 144 h in 3 x SC medium with exponential glucose feeding. PepB = Bovine peptone (10g/L). PepS = Soy peptone (10g/L). mean n = 3, with error bars indicating SD (* P<0.037, ** P<0.018. Welch t-test, one-sided).

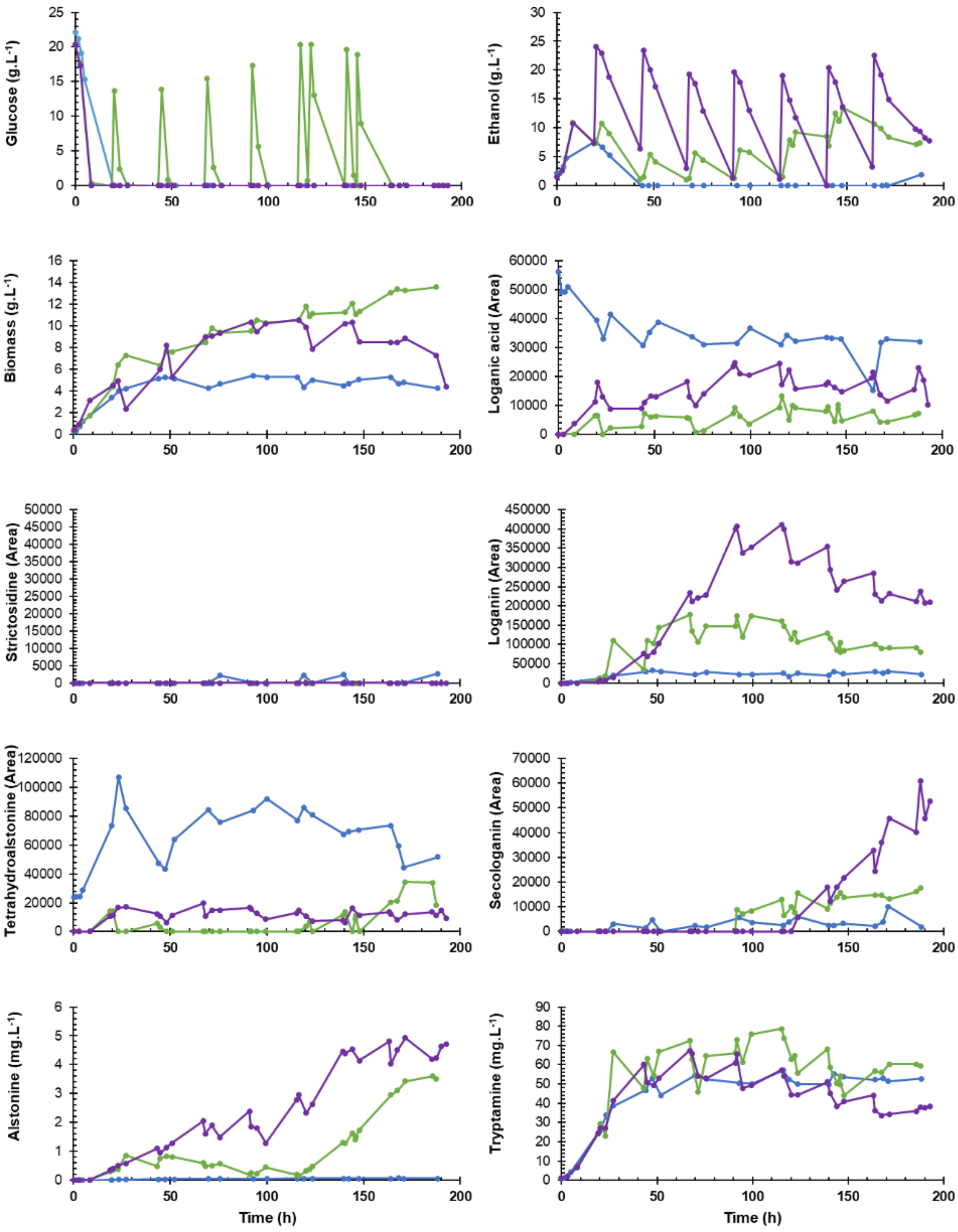

Supplementary Figure 6. Main extracellular metabolite levels and biomass during fed-batch fermentations at 2 liter scale. Sc112 under exponential glucose (blue line), FH144 on pulsated glucose (green line) and FH144 on pulsated ethanol (purple).

a

b

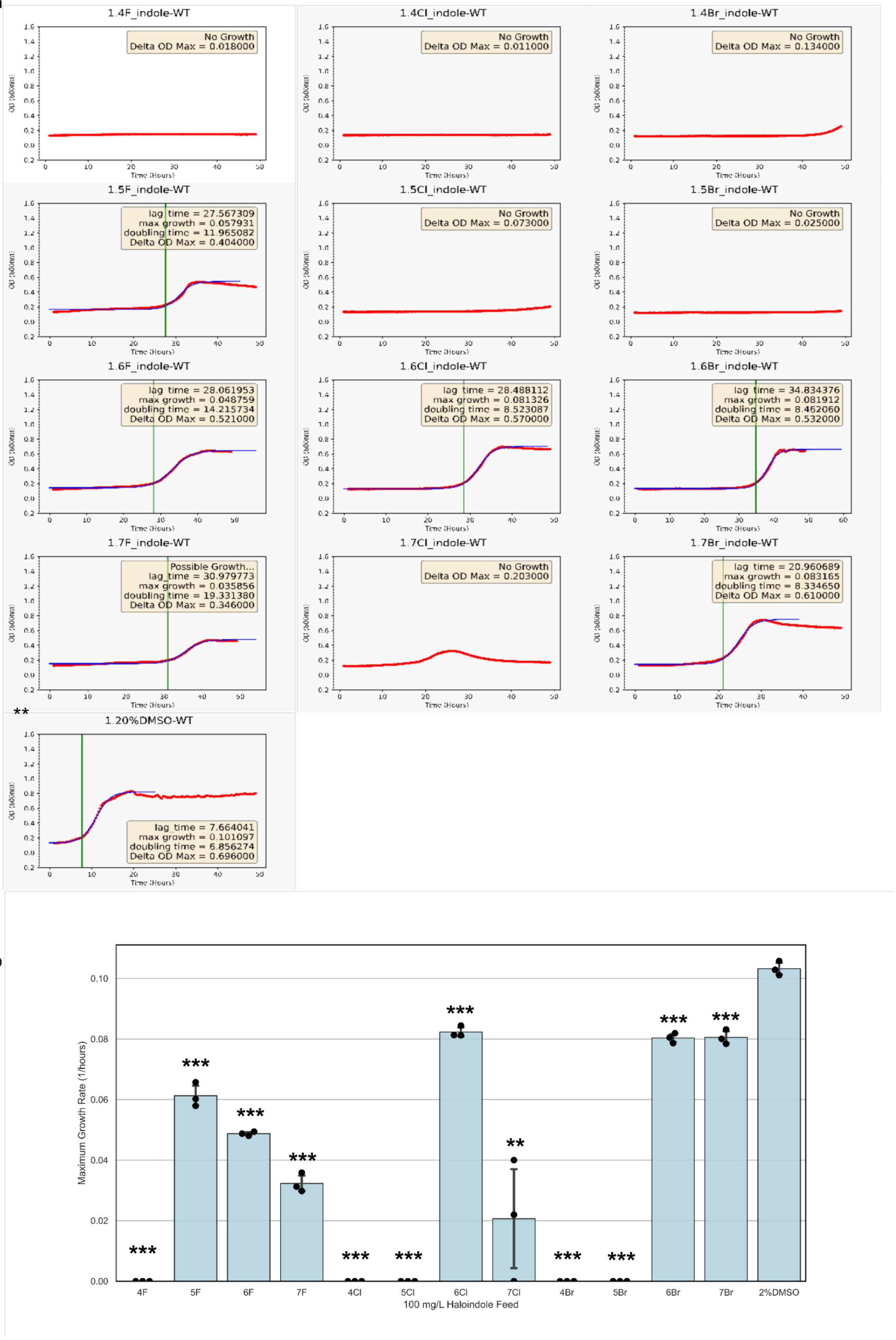
1.4F_indole-WT
No Growth
Delta OD Max = 0.018000
1.4Cl_indole-WT
No Growth
Delta OD Max = 0.011000
1.4Br_indole-WT
No Growth
Delta OD Max = 0.134000
1.5F_indole-WT
lag time = 27.567309
max growth = 0.057931
doubling time = 11.965082
Delta OD Max = 0.404000
1.5Cl_indole-WT
No Growth
Delta OD Max = 0.073000
1.5Br_indole-WT
No Growth
Delta OD Max = 0.025000
1.6F_indole-WT
lag time = 28.061953
max growth = 0.048759
doubling time = 14.215734
Delta OD Max = 0.521000
1.6Cl_indole-WT
lag time = 28.488112
max growth = 0.081326
doubling time = 8.523087
Delta OD Max = 0.570000
1.6Br_indole-WT
lag time = 34.834376
max growth = 0.081912
doubling time = 8.462060
Delta OD Max = 0.532000
1.7F_indole-WT
Possible Growth...
lag time = 30.979773
max growth = 0.035856
doubling time = 19.331380
Delta OD Max = 0.346000
1.7Cl_indole-WT
No Growth
Delta OD Max = 0.203000
1.7Br_indole-WT
lag time = 20.960689
max growth = 0.083165
doubling time = 8.334650
Delta OD Max = 0.610000
**
1.20%DMSO-WT
lag time = 7.664041
max growth = 0.101097
doubling time = 6.856274
Delta OD Max = 0.696000
Time (Hours)
Maximum Growth Rate (1/hours)
0.00
0.02
0.04
0.06
0.08
0.10
***
**
4F
5F
6F
7F
4Cl
5Cl
6Cl
7Cl
4Br
5Br
6Br
7Br
2%DMSO
100 mg/L Haloindole Feed

Supplementary Figure 7. Growth effect of halo-indoles on yeast. Triplicate cultures of 3xSC supplemented with 100 mg/L halo-indole 2% DMSO or a 2% DMSO control were inoculated with MIA-B0 and the OD600 was monitored for 48 hours. **a**) Representative growth curves for strain MIA-B0 supplemented with haloindoles. Data points are indicated in red, fitted growth curve in blue and the lag time in green. **b**) Mean calculated max growth rate for each triplicate, plotted with standard deviation and data points overlaid as black dots. Statistical significance of the difference from the 2% DMSO control was calculated using a two-tailed student's t-test and given above each bar; *** $p<0.001$, ** $p< 0.01$, * $p<0.05$.

a

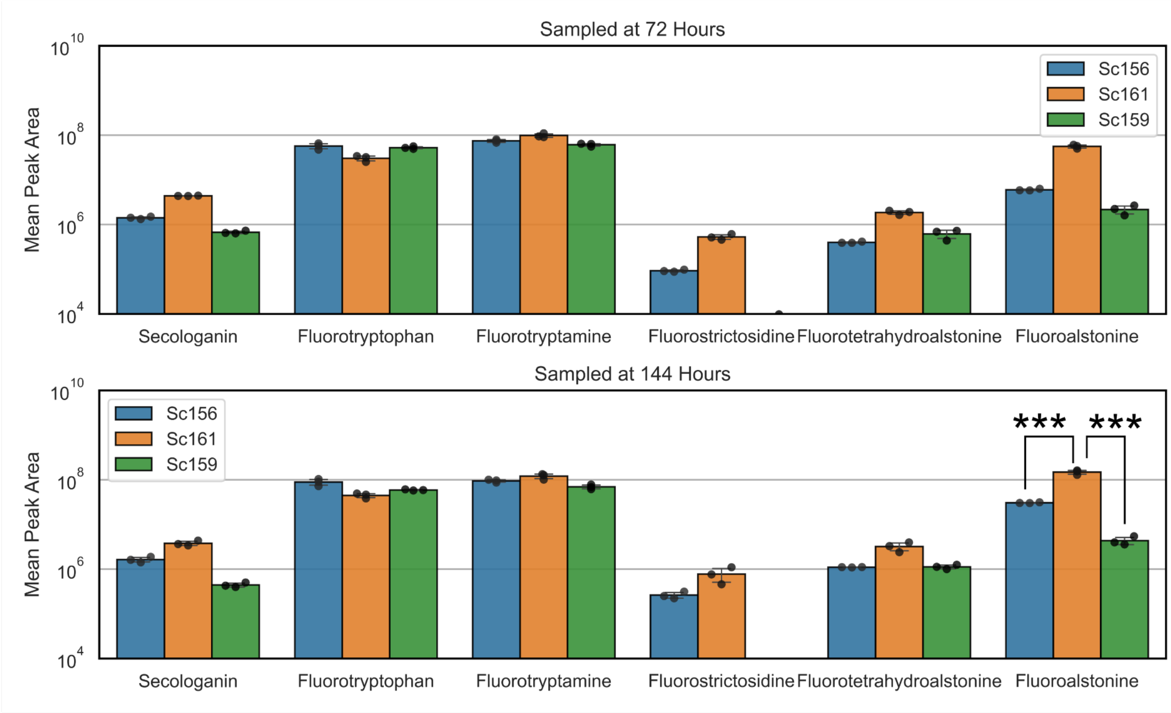

b

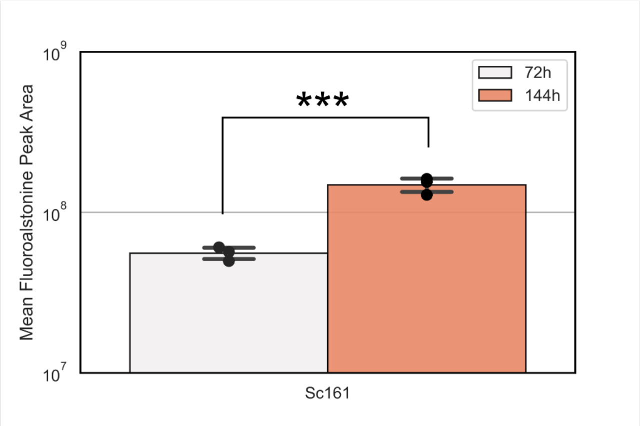

Supplementary Figure 8. Fluoro-alstonine production in strain Sc156, Sc159 and Sc161. a. Triplicate cultivation supplemented with 100 mg/L 7-fluoroindole and 0.25 mM secologanin of each strain were sampled at the 72 h and 144 h time points and analyzed with HRMS. Mean peak areas for fluorinated MIAs are plotted with standard deviation and data points overlaid as black dots. Statistical significance was calculated using the student's t-test; *** p<0.001, ** p< 0.01, *

$p<0.05$. **b.** Comparison of fluoroalstonine in broth of Sc161 cultivation at 72 h and 144 h.

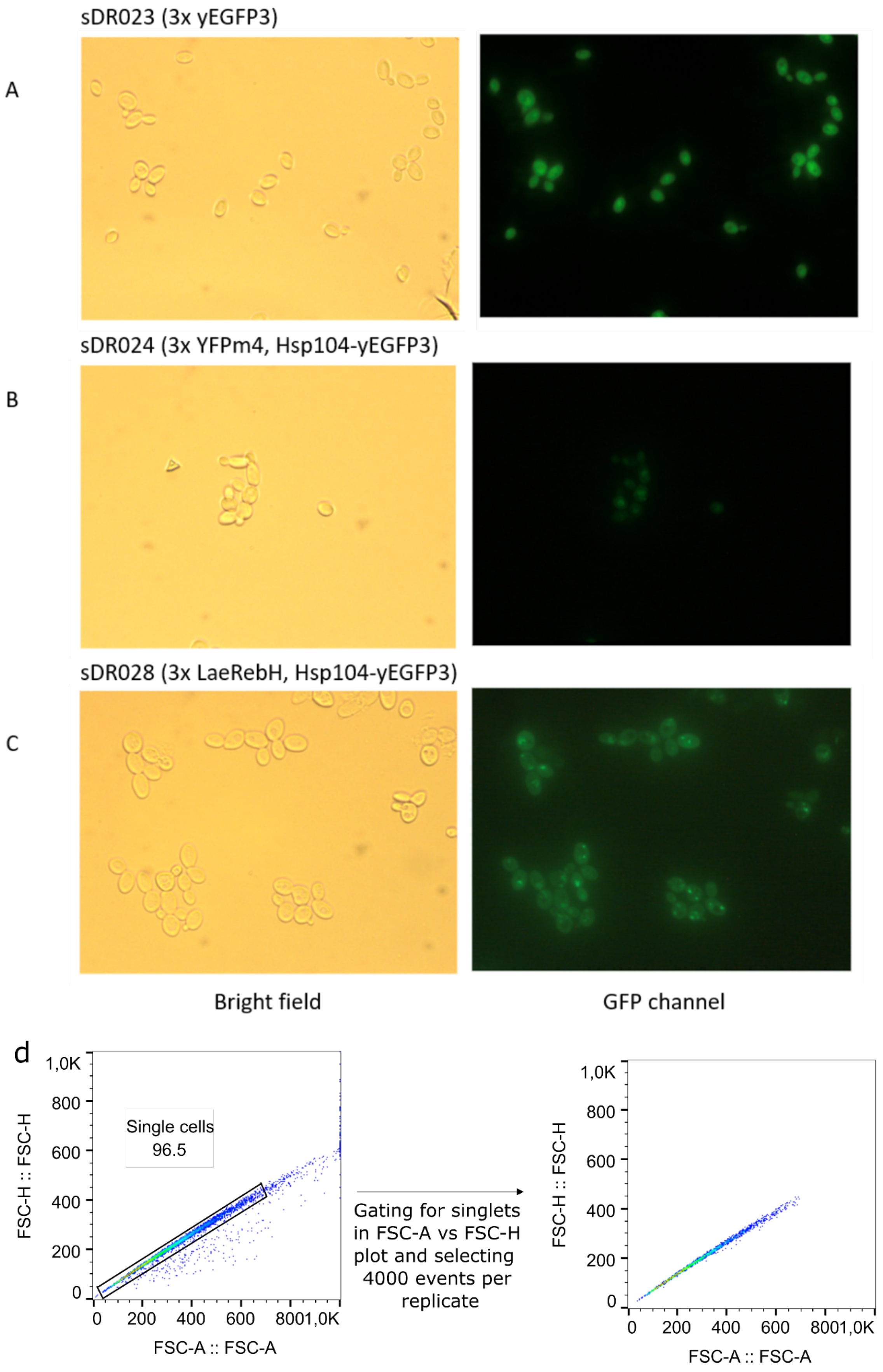
sDR023 (3x yEGFP3)
A
sDR024 (3x YFPm4, Hsp104-yEGFP3)
B
sDR028 (3x LaeRebH, Hsp104-yEGFP3)
C
Bright field
GFP channel
d
Single cells
96.5
FSC-H :: FSC-H
FSC-A :: FSC-A
0
200
400
600
800
1,0K
Gating for singlets in FSC-A vs FSC-H plot and selecting 4000 events per replicate

Supplementary Figure 9. Microscope images of *Lae*RebH expressing strains show aggregation in yeast. Strains expressing 3x copies of **a.** yEGFP3 (sDR023) **b.** aggregation-prone YFPm4 and Hsp104-yEGFP3 fusion (sDR024) and **c.** *Lae*RebH and Hsp104-yEGFP3 fusion (sDR028). **d.** Gating strategy used to obtain data presented in figure 5c. A single gating step was performed for singlets, and from each biological replicate, 4,000 events were exported for analysis.

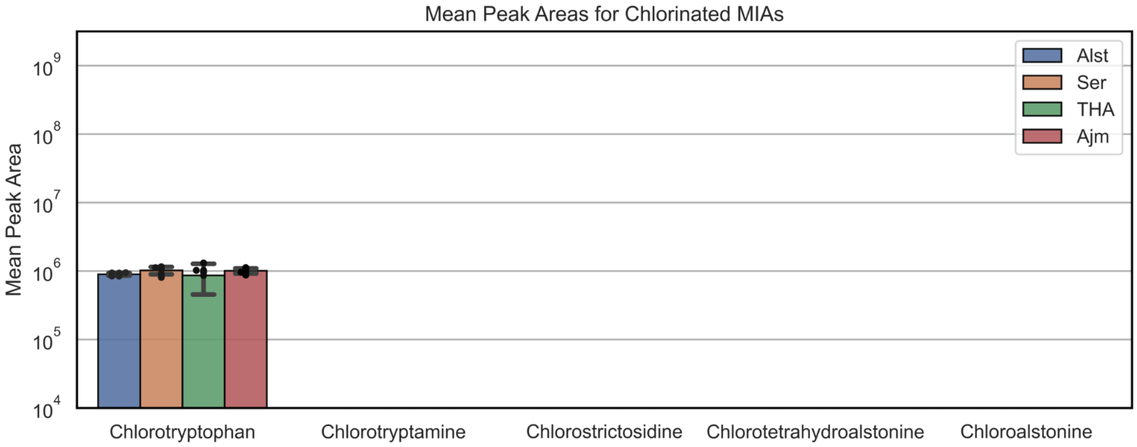

Supplementary Figure 10. *Lae*RebH is specific to tryptophan. ScH122 was cultivated in triplicate for 144 h in 3xSC 2% galactose supplemented with 300 mM NaCl and 10 mg/L alstonine (Als), serpentine (Serp), tetrahydroalstonine (THA) or ajmalicine (Ajm). Mean peak areas (n = 5) of chlorinated MIAs are plotted with standard deviation and individual data points overlaid.

Supplementary tables

Supplementary table. 1. Screen of alstonine synthase candidates in SC and yeast-extract/peptone/dextrose (YPD) cultivation medium.

Candidate	SC average (μg/L)	SC stdv	YPD average (μg/L)	YPD stdv
RteAS1	1.59	0.64	48.91	27.03

RteAS2	8.33	4.80	71.67	41.10
RteAS3	0.00	0.00	1.35	1.15
RteAS4	0.00	0.00	0.21	0.32
RteAS5	0.00	0.00	1.86	1.22
CroAS	0.00	0.00	0.16	0.40
CroAS2	0.00	0.00	46.88	15.94
RseSBE	0.00	0.00	0.13	0.11
GseSBE	0.37	0.20	7.29	4.43
CroAS_nat	0.00	0.00	0.76	0.76
CroAS2_nat	0.00	0.00	3.65	1.34
RseSBE_nat	0.23	0.26	0.73	0.47
GseSBE_nat	3.33	1.63	10.21	4.50
CroSS_nat	108.00	30.04	6641.35	1757.48
no AS (Ctrl)	0.19	0.31	3.02	2.27

Supplementary table. 2. Chemical standards used in this study.

**Chemical**	**CAS**	**Supplier**
caffeine (internal standard)	58-08-2	Sigma-Aldrich
loganic acid	22255-40-9	Carl Roth GmbH + Co (Germany)
loganin	18524-94-2	Santa Cruz Biotechnology (USA)
secologanin	19351-63-4	Sigma-Aldrich

tryptamine	61-54-1	Sigma-Aldrich
strictosidine	20824-29-7	PHYTOCONSULT (Netherlands) discontinued
tetrahydroalstonine	6474-90-4	Chengdu Push Bio-technology Co., Ltd. (China) discontinued
ajmalicine	483-04-5	Sigma-Aldrich
serpentine	58782-36-8	Toronto Research Chemicals
alstonine	642-18-2	Isolated from *Catharanthus roseus* ([2])
Indole	120-72-9	Sigma-Aldrich
4-Fluoroindole	387-43-9	Sigma-Aldrich
5-Fluoroindole	399-52-0	Sigma-Aldrich
6-Fluoroindole	399-51-9	Sigma-Aldrich
7-Fluoroindole	387-44-0	Sigma-Aldrich
4-Chloroindole	25235-85-2	Sigma-Aldrich
5-Chloroindole	17422-32-1	Sigma-Aldrich
6-Chloroindole	17422-33-2	Sigma-Aldrich
7-Chloroindole	53924-05-3	Sigma-Aldrich
4-Bromoindole	52488-36-5	Sigma-Aldrich
5-Bromoindole	10075-50-0	Sigma-Aldrich
6-Bromoindole	52415-29-9	Sigma-Aldrich
7-Bromoindole	51417-51-7	Sigma-Aldrich
4,5-Difluoroindole	247564-63-2	Sigma-Aldrich
4,6-Difluoroindole	199526-97-1	Sigma-Aldrich
4,7-Difluoroindole	247564-55-2	Sigma-Aldrich
5,6-Difluoroindole	169674-01-5	Sigma-Aldrich
5,7-Difluoroindole	301856-25-7	Sigma-Aldrich
6,7-Difluoroindole	271780-84-8	Sigma-Aldrich
(-)-Epinephrine	51-43-4	Sigma-Aldrich
Yohimbine hydrochloride	65-19-0	Sigma-Aldrich

Supplementary table 3. Genes in this study.

Name	Organism	Reference	Accession number

CroHYS	*Catharanthus roseus*	[3]	KU865325.1
CroTHAS1	*Catharanthus roseus*	[4]	KM524258
RseSGD	*Rauvolfia serpentina*	[5]	CAC83098.1
GseSBE	*Gelsemium sempervirens*	[2]	MF537712
CroAS2	*Catharanthus roseus*	This study	
CroAS	*Catharanthus roseus*	[2]	AHK60849.1
RseSBE	*Rauvolfia serpentina*	[2]	MF537711
RteAS-1	*Rauvolfia tetraphylla*	This study	
RteAS-2	*Rauvolfia tetraphylla*	This study	
RteAS-3	*Rauvolfia tetraphylla*	This study	
RteAS-4	*Rauvolfia tetraphylla*	This study	
RteAS-5	*Rauvolfia tetraphylla*	This study	
CroSS	*Catharanthus roseus*	[6]	MT829151
*lae*RebH	*Lechevalieria aerocolonigenes ATCC 39243*	[7]	CAC93722.1
*eco*SsuE	*Escherichia coli str. K-12 substr. MG1655*	[8]	NP_415457.1
*rgn*TDC	*Ruminococcus gnavus*	[9]	EC 4.1.1.28
ecoTrx	*Escherichia coli*	[10]	
ADRA2A_Nat	*Homo Sapiens*	[11]	HGNC:281
5HT2C_Nat	*Homo Sapiens*		HGNC:5295
NanoLuc	*Oplophorus gracilirostris*	[12]	Q9GV45

Supplementary table 4. Plasmids used and constructed in this study.

Description	Name	reference
2 µ, pESC-TRP1 USER vector	57	[13]
2 µ, pESC-URA3, gRNA-XI-2	6914	[1]

2 µ, pESC-LEU2, gRNA-IV-2-2	9661	[1]
2 µ, pESC-LEU2, gRNA-X-4	6901	[1]
2 µ, pESC-TRP1, gRNA-IV-1-1	11156	This study
2 µ, pESC-URA3, gRNA-X-2	6910	[1]
2 µ, pESC-URA3, XI-3	6915	[1]
2 µ, pESC-LEU2, gRNA-XII-4	6908	[1]
2 µ, pESC-LEU2, gRNA-sfGFP (GAACTGGACGGAGATGTAAA)	pFH 69	This study
pCMV, CMVenhancer-pCMV-5HT2C-tCMV : pSV40-f1ori-KanR	pFH78	This study
pINT-PLexO-NanoLuc-TCYC1	pFH 85	This study
pINT-XII-4, PCCW12-ADRA2A-TCYC1	12256	This study
pINT-X-2, PTDH3-RteAS1-TADH1	pBJL 37	This study
pINT-X-2, PTDH3-RteAS2-TADH1	pBJL 38	This study
pINT-X-2, PTDH3-RteAS3-TADH1	pBJL 40	This study
pINT-X-2, PTDH3-RteAS4-TADH1	pBJL 42	This study
pINT-X-2, PTDH3-RteAS5-TADH1	pBJL 44	This study
pINT-X-2, PTDH3-CroAS_nat-TADH1	pBJL 46	This study
pINT-X-2, PTDH3-RseSBE_nat-TADH1	pBJL 47	This study
pINT-X-2, PTDH3-GseSBE_nat-TADH1	pBJL 48	This study
pINT-X-2, PTDH3-CroAS2_nat-TADH1	pBJL 50	This study
pINT-X-2, PTDH3-CroAS2-TADH1	pBJL 51	This study
pINT-X-2, PTDH3-CroAS-TADH1	pBJL 52	This study
pINT-X-2, PTDH3-RseSBE-TADH1	pBJL 53	This study
pINT-X-2, PTDH3- GseSBE-TADH1	pBJL 54	This study
pINT-XI-3, PCCW12-RteAS2-TCYC1, PTDH3-GseSBE-TADH1	pBJL 57	This study
pINT-IV-2, PTEF1-RseSGD-TADH1,PPGK1-CroHYS-TCYC1	pBJL 81	This study
pINT-IV-1, PTEF1-CloCPR-TPRM9, PPGK1-CroCYB5-TVPS13	pBJL 87	This study
pINT-IV-1, PTEF1-RseCPR-TPRM9, PPGK1-CroCYB5-TVPS13	pBJL 88	This study
pINT-IV-1, PTEF1-OeuCPR-TPRM9, PPGK1-CroCYB5-TVPS13	pBJL 91	This study
pINT-IV-1, PTEF1-CpoCPR-TPRM9, PPGK1-CroCYB5-TVPS13	pBJL 92	This study
pINT-IV-1, PTEF1-CroCPR-TPRM9, PPGK1-CroCYB5-TVPS13	pBJL 93	This study
pINT-IV-1, PTEF1-AnnCPR-TPRM9, PPGK1-CroCYB5-TVPS13	pBJL 94	This study

pINT-IV-1, PTEF1-AthATR1-TPRM9, PPGK1-CroCYB5-TVPS13	pBJL 95	This study
pINT-IV-1, PTEF1-CacCPR-TPRM9, PPGK1-CroCYB5-TVPS13	pBJL 100	This study
pINT-IV-2, PTEF1-RseSGD-TADH1,PPGK1-CroTHAS1-TCYC1	pBJL 104	This study
pINT-XI-2, PTDH3-CroSS_nat-tADH1	pBJL 107	This study
CEN6/ARS4, pRS-HIS3,TEF1-RseSGD-TADH1, PGK1-CroTHAS1-TCYC	pBJL 168	This study
CEN6/ARS4, pRS-HIS3,TEF1-RseSGD-TADH1, PGK1-CroHYS-TCYC1	pBJL169	This study
2 μ, pESC-TRP1, TDH3-GseSBE_nat-TADH1	pBJL170	This study
2 μ, pESC-TRP1, TDH3-RteAS-2-TADH1	pBJL 171	This study
2 μ, pESC-LEU2, gRNA-CroCPR	pBJL 172	This study
pINT-XI-2, PGAL2-CroTHAS1-TCYC1, PTDH3-CroSS-TADH1	pBJL 174	This study
pINT-308a, PGAL1-CroSLS-TADH1, PTEF1-INO2-TCYC1	pBJL 177	This study
pINT-IV-2, pGAL1-RebH-tADH1, pGAL10-SsuE-tCYC1	pSAB1	This study
pINT-XI-3, PTEF1-AanCPR-tCYC1	pFH 41	This study
pINT-XI-3, PTEF1-CloCPR-tCYC1	pFH 42	This study
pINT-IV-1, PTEF1-RseSGD-TADH1,PPGK1-CroTHAS1-TCYC1	pFH 43	This study
pINT-IV-1, PTEF1-RseSGD-TADH1,PPGK1-CroHYS-TCYC1	pFH 44	This study
pINT-IV-2, pGAL1-Trx-RebH-tADH1, pGAL10-SsuE-tCYC1	pSAB2	This study
pINT-IV-2, pGAL1-T7B9-RebH-tADH1, pGAL10-SsuE-tCYC1	pSAB3	This study
pINT-308a, pGAL1-RebH-tADH1, pGAL10-SsuE-tCYC1	pSAB4	This study
pINT-511b, pGAL1-RebH-tADH1, pGAL10-SsuE-tCYC1	pSAB5	This study
TEF1p-Cas9-CYC1t	pcfb1767	[14]
pINT-X-4:4xUAS-SSA1p-365:mKate2-CYC1t	pYR70	[15]
SNR52p-X-4_gRNA-SUP4	pcfb6901	Zhang *et al.* unpublished
SNR52p-LP3_gRNA-SUP4	pDR062	This work
SNR52p-HSP104_gRNA-SUP4	pDR065	This work
LP3: GAL1p-yEGFP-ENO1t	pDR048	This work
LP3: GAL1p-YFPm4-ENO1t	pDR049	This work
LP3: GAL1p-RebH-ENO1t	pDR053	This work
Hsp104:yEGFP3	fDR176	This work

Supplementary table 5. Strains used and constructed in this study. All the genes were codon optimized, except when assigned as "_nat".

Name	Genotype	Reference
yWS2267 ,	**BY4741** - MAT**a**, sst2Δ0, far1Δ0, bar1Δ0, ste2Δ0, ste12Δ0, gpa1Δ0, ste3Δ0, mf(alpha)1Δ0, mf(alpha)2Δ0, mfa1Δ0, mfa2Δ0, gpr1Δ0, gpa2Δ0, LexO(6x)-pLEU2m-sfGFP-tTDH1-pCCW12-STE2-tSSA1-pPGK1-GPA1-tENO2-pRAD27-LexA-PRD-tENO1-URA3,	[16]
Sc237	**BY4741** - MAT**a**, sst2Δ0, far1Δ0, bar1Δ0, ste2Δ0, ste12Δ0, gpa1Δ0, ste3Δ0, mf(alpha)1Δ0, mf(alpha)2Δ0, mfa1Δ0, mfa2Δ0, gpr1Δ0, gpa2Δ0, LexO(6x)-pLEU2m-**NanoLuc**-tTDH1-pCCW12-STE2-tSSA1-pPGK1-GPA1-tENO2-pRAD27-LexA-PRD-tENO1-URA3,	This study
Sc272	**BY4741** - MAT**a**, sst2Δ0, far1Δ0, bar1Δ0, ste2Δ0, ste12Δ0, gpa1Δ0, ste3Δ0, mf(alpha)1Δ0, mf(alpha)2Δ0, mfa1Δ0, mfa2Δ0, gpr1Δ0, gpa2Δ0, LexO(6x)-pLEU2m-**NanoLuc**-tTDH1-pCCW12-STE2-tSSA1-pPGK1-GPA1-tENO2-pRAD27-LexA-PRD-tENO1-URA3, PCCW12-ADRA2A-TCYC1	This study
MIA-B0	MATa; his3D1; leu2-3_112; ura3-52; trp1-289; pTEF1-SpCas9-tCYC1,	[1]
MIA-CM-3	MAT**a**, his3D1, leu2-3_112, ura3-52, trp1-289, atf1Δ oye2Δ adh6Δ oye3Δ ari1Δ, PTEF1-SpyCas9-TCYC1, PTEF1-CroCPR-TPRM9, PPGK1-CroCYB5-TIDP1, PMLS1-AgrGPPS2-TVPS13, PFBA1-GgaFPSN144W-TIDP1, PPGK1-IDI1-TPRM9, PTDH3-tHMG1-TADH1, PICL1-ERG20F96W, N127WtCroGES-TCYC1, PPGK1-CroTDC-TPRM9, PTDH3-CroG8H-TADH1, PPGK1-Vmi8HGO-A -TADH1,PFBA1-NcaISY-TCYC1, PTEF1-NcaMLPLA-TADH1, PTEF2-CroIO-TCYC1, PFBA1-CroADH2-TCPS1,PPGK1-Cro7DLGT-TVPS13, PTEF2-Cro7DLH-TCYC1, PTDH3-CroLAMT-TADH1, PTPI1-CroSLS-TIDP1,PFBA1-CroSTR-TPRM9	[1]
MIA-CM5	MAT**a**, his3D1, leu2-3_112, ura3-52, trp1-289, atf1Δ oye2Δ adh6Δ oye3Δ ari1Δ,, PTEF1-SpyCas9-TCYC1, PTEF1-CroCPR-TPRM9, PPGK1-CroCYB5-TIDP1, **PTEF2-AgrGPPS2-TVPS13**, PFBA1-GgaFPSN144W-TIDP1, PPGK1-IDI1-TPRM9, PTDH3-tHMG1-TADH1, **PCCW12-ERG20F96W**, N127WtCroGES-TCYC1, PPGK1-CroTDC-TPRM9, PTDH3-CroG8H-TADH1, PPGK1-Vmi8HGO-A -TADH1,PFBA1-NcaISY-TCYC1, PTEF1-NcaMLPLA-TADH1, PTEF2-CroIO-TCYC1, PFBA1-CroADH2-TCPS1,PPGK1-Cro7DLGT-TVPS13, PTEF2-Cro7DLH-TCYC1, PTDH3-CroLAMT-TADH1, PTPI1-CroSLS-TIDP1,PFBA1-CroSTR-TPRM9.	[1]
MIA-CM10	MAT**a**, his3D1, leu2-3_112, ura3-52, trp1-289, atf1Δ oye2Δ adh6Δ oye3Δ ari1Δ, PTEF1-SpyCas9-TCYC1, PTEF1-CroCPR-TPRM9, PPGK1-CroCYB5-TIDP1, PMLS1-AgrGPPS2-TVPS13, PFBA1-GgaFPSN144W-TIDP1, PPGK1-IDI1-TPRM9, PTDH3-tHMG1-TADH1, PICL1-ERG20F96W, N127WtCroGES-TCYC1, PGAL1-**RgnTDC**-TPRM9, PTDH3-CroG8H-TADH1, PPGK1-Vmi8HGO-A -TADH1,PFBA1-NcaISY-TCYC1, PTEF1-NcaMLPLA-TADH1, PTEF2-CroIO-TCYC1, PFBA1-CroADH2-TCPS1,PPGK1-Cro7DLGT-TVPS13, PTEF2-Cro7DLH-TCYC1, PTDH3-CroLAMT-TADH1, PTPI1-CroSLS-TIDP1,PFBA1-CroSTR-TPRM9	This study
Sc67	MAT**a**, his3D1, leu2-3_112, ura3-52, trp1-289, atf1Δ oye2Δ adh6Δ oye3Δ ari1Δ, PTEF1-SpyCas9-TCYC1, PTEF1-CroCPR-TPRM9, PPGK1-CroCYB5-TIDP1, PMLS1-AgrGPPS2-TVPS13, PFBA1-GgaFPSN144W-TIDP1, PPGK1-IDI1-TPRM9, PTDH3-tHMG1-TADH1, PICL1-ERG20F96W, N127WtCroGES-TCYC1, PPGK1-CroTDC-	This study

	TPRM9, PTDH3-CroG8H-TADH1, PPGK1-Vmi8HGO-A -TADH1,PFBA1-NcaISY-TCYC1, PTEF1-NcaMLPLA-TADH1, PTEF2-CroIO-TCYC1, PFBA1-CroADH2-TCPS1,PPGK1-Cro7DLGT-TVPS13, PTEF2-Cro7DLH-TCYC1, PTDH3-CroLAMT-TADH1, PTPI1-CroSLS-TIDP1,PFBA1-CroSTR-TPRM9, PTEF1-RseSGD-TADH1,PGK1-CroTHAS1-TCYC1	
Sc76	MAT**a**, his3D1, leu2-3_112, ura3-52, trp1-289, atf1Δ oye2Δ adh6Δ oye3Δ ari1Δ, PTEF1-SpyCas9-TCYC1, PTEF1-CroCPR-TPRM9, PPGK1-CroCYB5-TIDP1, PMLS1-AgrGPPS2-TVPS13, PFBA1-GgaFPSN144W-TIDP1, PPGK1-IDI1-TPRM9, PTDH3-tHMG1-TADH1, PICL1-ERG20F96W, N127WtCroGES-TCYC1, PPGK1-CroTDC-TPRM9, PTDH3-CroG8H-TADH1, PPGK1-Vmi8HGO-A -TADH1,PFBA1-NcaISY-TCYC1, PTEF1-NcaMLPLA-TADH1, PTEF2-CroIO-TCYC1, PFBA1-CroADH2-TCPS1,PPGK1-Cro7DLGT-TVPS13, PTEF2-Cro7DLH-TCYC1, PTDH3-CroLAMT-TADH1, PTPI1-CroSLS-TIDP1,PFBA1-CroSTR-TPRM9, PTEF1-RseSGD-TADH1, PGK1-CroHYS-TCYC	This study
Sc77	MAT**a**, his3D1, leu2-3_112, ura3-52, trp1-289, atf1Δ oye2Δ adh6Δ oye3Δ ari1Δ, PTEF1-SpyCas9-TCYC1, PTEF1-CroCPR-TPRM9, PPGK1-CroCYB5-TIDP1, PMLS1-AgrGPPS2-TVPS13, PFBA1-GgaFPSN144W-TIDP1, PPGK1-IDI1-TPRM9, PTDH3-tHMG1-TADH1, PICL1-ERG20F96W, N127WtCroGES-TCYC1, PPGK1-CroTDC-TPRM9, PTDH3-CroG8H-TADH1, PPGK1-Vmi8HGO-A -TADH1,PFBA1-NcaISY-TCYC1, PTEF1-NcaMLPLA-TADH1, PTEF2-CroIO-TCYC1, PFBA1-CroADH2-TCPS1,PPGK1-Cro7DLGT-TVPS13, PTEF2-Cro7DLH-TCYC1, PTDH3-CroLAMT-TADH1, PTPI1-CroSLS-TIDP1,PFBA1-CroSTR-TPRM9, PTEF1-RseSGD-TADH1,PGK1-CroTHAS1-TCYC1, PTDH3-RteAS-2-TADH1	This study
Sc78	MAT**a**, his3D1, leu2-3_112, ura3-52, trp1-289, atf1Δ oye2Δ adh6Δ oye3Δ ari1Δ, PTEF1-SpyCas9-TCYC1, PTEF1-CroCPR-TPRM9, PPGK1-CroCYB5-TIDP1, PMLS1-AgrGPPS2-TVPS13, PFBA1-GgaFPSN144W-TIDP1, PPGK1-IDI1-TPRM9, PTDH3-tHMG1-TADH1, PICL1-ERG20F96W, N127WtCroGES-TCYC1, PPGK1-CroTDC-TPRM9, PTDH3-CroG8H-TADH1, PPGK1-Vmi8HGO-A -TADH1,PFBA1-NcaISY-TCYC1, PTEF1-NcaMLPLA-TADH1, PTEF2-CroIO-TCYC1, PFBA1-CroADH2-TCPS1,PPGK1-Cro7DLGT-TVPS13, PTEF2-Cro7DLH-TCYC1, PTDH3-CroLAMT-TADH1, PTPI1-CroSLS-TIDP1,PFBA1-CroSTR-TPRM9, PTEF1-RseSGD-TADH1,PGK1-CroTHAS1-TCYC1, PTDH3-GseSBE-TADH1	This study
Sc79	MAT**a**, his3D1, leu2-3_112, ura3-52, trp1-289, atf1Δ oye2Δ adh6Δ oye3Δ ari1Δ, PTEF1-SpyCas9-TCYC1, PTEF1-CroCPR-TPRM9, PPGK1-CroCYB5-TIDP1, PMLS1-AgrGPPS2-TVPS13, PFBA1-GgaFPSN144W-TIDP1, PPGK1-IDI1-TPRM9, PTDH3-tHMG1-TADH1, PICL1-ERG20F96W, N127WtCroGES-TCYC1, PPGK1-CroTDC-TPRM9, PTDH3-CroG8H-TADH1, PPGK1-Vmi8HGO-A -TADH1,PFBA1-NcaISY-TCYC1, PTEF1-NcaMLPLA-TADH1, PTEF2-CroIO-TCYC1, PFBA1-CroADH2-TCPS1,PPGK1-Cro7DLGT-TVPS13, PTEF2-Cro7DLH-TCYC1, PTDH3-CroLAMT-TADH1, PTPI1-CroSLS-TIDP1,PFBA1-CroSTR-TPRM9, PTEF1-RseSGD-TADH1,PGK1-CroTHAS1-TCYC1, PTDH3-GseSBE-TADH1, PCCW12-RteAS2-TCYC1	This study
Sc80	MAT**a**, his3D1, leu2-3_112, ura3-52, trp1-289, atf1Δ oye2Δ adh6Δ oye3Δ ari1Δ, PTEF1-SpyCas9-TCYC1, PTEF1-CroCPR-TPRM9, PPGK1-CroCYB5-TIDP1, PTEF2-AgrGPPS2-TVPS13, PFBA1-GgaFPSN144W-TIDP1, PPGK1-IDI1-TPRM9, PTDH3-tHMG1-TADH1, pCCW12-ERG20F96W, N127WtCroGES-TCYC1, PPGK1-CroTDC-TPRM9, PTDH3-CroG8H-TADH1, PPGK1-Vmi8HGO-A -	This study

	TADH1,PFBA1-NcaISY-TCYC1, PTEF1-NcaMLPLA-TADH1, PTEF2-CroIO-TCYC1, PFBA1-CroADH2-TCPS1,PPGK1-Cro7DLGT-TVPS13, PTEF2-Cro7DLH-TCYC1, PTDH3-CroLAMT-TADH1, PTPI1-CroSLS-TIDP1,PFBA1-CroSTR-TPRM9, PTEF1-RseSGD-TADH1,PGK1-CroTHAS1-TCYC1, PTDH3-RteAS-2-TADH1	
Sc81	MAT**a**, his3D1, leu2-3_112, ura3-52, trp1-289, atf1Δ oye2Δ adh6Δ oye3Δ ari1Δ, PTEF1-SpyCas9-TCYC1, PTEF1-CroCPR-TPRM9, PPGK1-CroCYB5-TIDP1, PTEF2-AgrGPPS2-TVPS13, PFBA1-GgaFPSN144W-TIDP1, PPGK1-IDI1-TPRM9, PTDH3-tHMG1-TADH1, pCCW12-ERG20F96W, N127WtCroGES-TCYC1, PPGK1-CroTDC-TPRM9, PTDH3-CroG8H-TADH1, PPGK1-Vmi8HGO-A -TADH1,PFBA1-NcaISY-TCYC1, PTEF1-NcaMLPLA-TADH1, PTEF2-CroIO-TCYC1, PFBA1-CroADH2-TCPS1,PPGK1-Cro7DLGT-TVPS13, PTEF2-Cro7DLH-TCYC1, PTDH3-CroLAMT-TADH1, PTPI1-CroSLS-TIDP1,PFBA1-CroSTR-TPRM9, PTEF1-RseSGD-TADH1,PGK1-CroTHAS1-TCYC1, PTDH3-GseSBE-TADH1	This study
Sc83	MAT**a**; his3D1; leu2-3_112; ura3-52; trp1-289; PTEF1-SpCas9-TCYC1, PGK1-CroCYB5-TVPS13, PFBA1-CroSTR- TPRM9, TEF1-RseSGD-TADH1,PPGK1-CroTHAS1-TCYC1, PTDH3-RteAS-2-TADH1	This study
Sc84	MAT**a**; his3D1; leu2-3_112; ura3-52; trp1-289; PTEF1-SpCas9-TCYC1, PGK1-CroCYB5-TVPS13, PFBA1-CroSTR- TPRM9, TEF1-RseSGD-TADH1,PPGK1-CroTHAS1-TCYC1, PTDH3-GseSBE-TADH1	This study
Sc85	MAT**a**, his3D1, leu2-3_112, ura3-52, trp1-289, atf1Δ oye2Δ adh6Δ oye3Δ ari1Δ, PTEF1-SpyCas9-TCYC1, PTEF1-CroCPR-TPRM9, PPGK1-CroCYB5-TIDP1, PMLS1-AgrGPPS2-TVPS13, PFBA1-GgaFPSN144W-TIDP1, PPGK1-IDI1-TPRM9, PTDH3-tHMG1-TADH1, PICL1-ERG20F96W, N127WtCroGES-TCYC1, PPGK1-CroTDC-TPRM9, PTDH3-CroG8H-TADH1, PPGK1-Vmi8HGO-A -TADH1,PFBA1-NcaISY-TCYC1, PTEF1-NcaMLPLA-TADH1, PTEF2-CroIO-TCYC1, PFBA1-CroADH2-TCPS1,PPGK1-Cro7DLGT-TVPS13, PTEF2-Cro7DLH-TCYC1, PTDH3-CroLAMT-TADH1, PTPI1-CroSLS-TIDP1,PFBA1-CroSTR-TPRM9, PTEF1-RseSGD-TADH1, PGK1-CroHYS-TCYC1, PTDH3-CroSS_nat-TADH1	This study
Sc86	MAT**a**; his3D1; leu2-3_112; ura3-52; trp1-289; PTEF1-SpCas9-TCYC1, PGK1-CroCYB5-TVPS13, PTEF1-CroCPR-tCYC1, PFBA1-CroSTR-TPRM9, PTEF1-RseSGD-TADH1,PPGK1-CroTHAS1-TCYC1	This study
Sc87	MAT**a**; his3D1; leu2-3_112; ura3-52; trp1-289; PTEF1-SpCas9-TCYC1, PGK1-CroCYB5-TVPS13, PTEF1-CroCPR-tCYC1, PFBA1-CroSTR-TPRM9, PTEF1-RseSGD-TADH1,PPGK1-CroTHAS1-TCYC1, PTDH3-RteAS1-TADH1	This study
Sc88	MAT**a**; his3D1; leu2-3_112; ura3-52; trp1-289; PTEF1-SpCas9-TCYC1, PGK1-CroCYB5-TVPS13, PTEF1-CroCPR-tCYC1, PFBA1-CroSTR-TPRM9, PTEF1-RseSGD-TADH1,PPGK1-CroTHAS1-TCYC1, PTDH3-RteAS2-TADH1	This study
Sc90	MAT**a**; his3D1; leu2-3_112; ura3-52; trp1-289; PTEF1-SpCas9-TCYC1, PGK1-CroCYB5-TVPS13, PTEF1-CroCPR-tCYC1, PFBA1-CroSTR-TPRM9, PTEF1-RseSGD-TADH1,PPGK1-CroTHAS1-TCYC1, PTDH3-RteAS3-TADH1	This study
Sc92	MAT**a**; his3D1; leu2-3_112; ura3-52; trp1-289; PTEF1-SpCas9-TCYC1, PGK1-CroCYB5-TVPS13, PTEF1-CroCPR-tCYC1, PFBA1-CroSTR-TPRM9, PTEF1-RseSGD-TADH1,PPGK1-CroTHAS1-TCYC1, PTDH3-RteAS4-TADH1	This study

Sc94	MAT**a**; his3D1; leu2-3_112; ura3-52; trp1-289; PTEF1-SpCas9-TCYC1, PGK1-CroCYB5-TVPS13, PTEF1-CroCPR-tCYC1, PFBA1-CroSTR-TPRM9, PTEF1-RseSGD-TADH1,PPGK1-CroTHAS1-TCYC1, PTDH3-RteAS5-TADH1	This study
Sc96	MAT**a**; his3D1; leu2-3_112; ura3-52; trp1-289; PTEF1-SpCas9-TCYC1, PGK1-CroCYB5-TVPS13, PTEF1-CroCPR-tCYC1, PFBA1-CroSTR-TPRM9, PTEF1-RseSGD-TADH1,PPGK1-CroTHAS1-TCYC1, PTDH3-CroAS_nat -TADH1	This study
Sc97	MAT**a**; his3D1; leu2-3_112; ura3-52; trp1-289; PTEF1-SpCas9-TCYC1, PGK1-CroCYB5-TVPS13, PTEF1-CroCPR-tCYC1, PFBA1-CroSTR-TPRM9, PTEF1-RseSGD-TADH1,PPGK1-CroTHAS1-TCYC1, PTDH3-RseSBE_nat-TADH1	This study
Sc98	MAT**a**; his3D1; leu2-3_112; ura3-52; trp1-289; PTEF1-SpCas9-TCYC1, PGK1-CroCYB5-TVPS13, PTEF1-CroCPR-tCYC1, PFBA1-CroSTR-TPRM9, PTEF1-RseSGD-TADH1,PPGK1-CroTHAS1-TCYC1, PTDH3-GseSBE_nat-TADH1	This study
Sc100	MAT**a**; his3D1; leu2-3_112; ura3-52; trp1-289; PTEF1-SpCas9-TCYC1, PGK1-CroCYB5-TVPS13, PTEF1-CroCPR-tCYC1, PFBA1-CroSTR-TPRM9, PTEF1-RseSGD-TADH1,PPGK1-CroTHAS1-TCYC1, PTDH3-CroAS2_nat-TADH1	This study
Sc101	MAT**a**; his3D1; leu2-3_112; ura3-52; trp1-289; PTEF1-SpCas9-TCYC1, PGK1-CroCYB5-TVPS13, PTEF1-CroCPR-tCYC1, PFBA1-CroSTR-TPRM9, PTEF1-RseSGD-TADH1,PPGK1-CroTHAS1-TCYC1, PTDH3-CroAS2-TADH1	This study
Sc102	MAT**a**; his3D1; leu2-3_112; ura3-52; trp1-289; PTEF1-SpCas9-TCYC1, PGK1-CroCYB5-TVPS13, PTEF1-CroCPR-tCYC1, PFBA1-CroSTR-TPRM9, PTEF1-RseSGD-TADH1,PPGK1-CroTHAS1-TCYC1, PTDH3-CroAS-TADH1	This study
Sc103	MAT**a**; his3D1; leu2-3_112; ura3-52; trp1-289; PTEF1-SpCas9-TCYC1, PGK1-CroCYB5-TVPS13, PTEF1-CroCPR-tCYC1, PFBA1-CroSTR-TPRM9, PTEF1-RseSGD-TADH1,PPGK1-CroTHAS1-TCYC1, PTDH3-RseSBE-TADH1	This study
Sc104	MAT**a**; his3D1; leu2-3_112; ura3-52; trp1-289; PTEF1-SpCas9-TCYC1, PGK1-CroCYB5-TVPS13, PTEF1-CroCPR-tCYC1, PFBA1-CroSTR-TPRM9, PTEF1-RseSGD-TADH1,PPGK1-CroTHAS1-TCYC1, PTDH3-GseSBE-TADH1	This study
Sc112	MAT**a**, his3D1, leu2-3_112, ura3-52, trp1-289, atf1Δ oye2Δ adh6Δ oye3Δ ari1Δ, PTEF1-SpyCas9-TCYC1, PTEF1-CroCPR-TPRM9, PPGK1-CroCYB5-TIDP1, PMLS1-AgrGPPS2-TVPS13, PFBA1-GgaFPSN144W-TIDP1, PPGK1-IDI1-TPRM9, PTDH3-tHMG1-TADH1, PICL1-ERG20F96W, N127WtCroGES-TCYC1, PPGK1-CroTDC-TPRM9, PTDH3-CroG8H-TADH1, PPGK1-Vmi8HGO-A -TADH1,PFBA1-NcaISY-TCYC1, PTEF1-NcaMLPLA-TADH1, PTEF2-CroIO-TCYC1, PFBA1-CroADH2-TCPS1,PPGK1-Cro7DLGT-TVPS13, PTEF2-Cro7DLH-TCYC1, PTDH3-CroLAMT-TADH1, PTPI1-CroSLS-TIDP1,PFBA1-CroSTR-TPRM9, PTEF1-RseSGD-TADH1,PGK1-CroTHAS1-TCYC1, PTDH3-CroSS_nat-TADH1	This study
Sc154	MAT**a**, his3D1, leu2-3_112, ura3-52, trp1-289, atf1Δ oye2Δ adh6Δ oye3Δ ari1Δ, PTEF1-SpyCas9-TCYC1, PTEF1-CroCPR-TPRM9, PPGK1-CroCYB5-TIDP1, PMLS1-AgrGPPS2-TVPS13, PFBA1-GgaFPSN144W-TIDP1, PPGK1-IDI1-TPRM9, PTDH3-tHMG1-TADH1,	This study

	PICL1-ERG20F96W, N127WtCroGES-TCYC1, **PGAL1-RgnTDC**-TPRM9, PTDH3-CroG8H-TADH1, PPGK1-Vmi8HGO-A -TADH1,PFBA1-NcaISY-TCYC1, PTEF1-NcaMLPLA-TADH1, PTEF2-CroIO-TCYC1, PFBA1-CroADH2-TCPS1,PPGK1-Cro7DLGT-TVPS13, PTEF2-Cro7DLH-TCYC1, PTDH3-CroLAMT-TADH1, PTPI1-CroSLS-TIDP1,PFBA1-CroSTR-TPRM9,**PTEF1-RseSGD-TADH1,PGK1-CroTHAS1-TCYC1, PTDH3-CroSS-TADH1**	
Sc156	MAT**a**, his3D1, leu2-3_112, ura3-52, trp1-289, atf1Δ oye2Δ adh6Δ oye3Δ ari1Δ, PTEF1-SpyCas9-TCYC1, PTEF1-CroCPR-TPRM9, PPGK1-CroCYB5-TIDP1, PMLS1-AgrGPPS2-TVPS13, PFBA1-GgaFPSN144W-TIDP1, PPGK1-IDI1-TPRM9, PTDH3-tHMG1-TADH1, PICL1-ERG20F96W, N127WtCroGES-TCYC1, **PGAL1-RgnTDC**-TPRM9, PTDH3-CroG8H-TADH1, PPGK1-Vmi8HGO-A -TADH1,PFBA1-NcaISY-TCYC1, PTEF1-NcaMLPLA-TADH1, PTEF2-CroIO-TCYC1, PFBA1-CroADH2-TCPS1,PPGK1-Cro7DLGT-TVPS13, PTEF2-Cro7DLH-TCYC1, PTDH3-CroLAMT-TADH1, PTPI1-CroSLS-TIDP1,PFBA1-CroSTR-TPRM9,PTEF1-RseSGD-TADH1,**PGK1-CroTHAS1**-TCYC1<br>**PGAL2-CroTHAS1**-TCYC1, PTDH3-CroSS-TADH1	This study
Sc157	MAT**a**; his3D1; leu2-3_112; ura3-52; trp1-289; PTEF1-SpCas9-TCYC1, PGK1-CroCYB5-TVPS13, PTEF1-CroCPR-tCYC1, PFBA1-CroSTR-TPRM9, PTEF1-RseSGD-TADH1,PPGK1-CroTHAS1-TCYC1, PTDH3-CroSS-TADH1	This study
Sc159	MAT**a**, his3D1, leu2-3_112, ura3-52, trp1-289, atf1Δ oye2Δ adh6Δ oye3Δ ari1Δ, PTEF1-SpyCas9-TCYC1, PTEF1-CroCPR-TPRM9, PPGK1-CroCYB5-TIDP1, PMLS1-AgrGPPS2-TVPS13, PFBA1-GgaFPSN144W-TIDP1, PPGK1-IDI1-TPRM9, PTDH3-tHMG1-TADH1, PICL1-ERG20F96W, N127WtCroGES-TCYC1, **PGAL1-RgnTDC**-TPRM9, PTDH3-CroG8H-TADH1, PPGK1-Vmi8HGO-A -TADH1,PFBA1-NcaISY-TCYC1, PTEF1-NcaMLPLA-TADH1, PTEF2-CroIO-TCYC1, PFBA1-CroADH2-TCPS1,PPGK1-Cro7DLGT-TVPS13, PTEF2-Cro7DLH-TCYC1, PTDH3-CroLAMT-TADH1, PTPI1-CroSLS-TIDP1,PFBA1-CroSTR-TPRM9,PTEF1-RseSGD-TADH1,PGK1-CroTHAS1-TCYC1, **PGAL2-CroTHAS1**-TCYC1, PTDH3-CroSS-TADH1, **PGAL1-CroSLS**-TADH1, **PTEF1-INO2**-TCYC1	This study
Sc161	MAT**a**, his3D1, leu2-3_112, ura3-52, trp1-289, atf1Δ oye2Δ adh6Δ oye3Δ ari1Δ, **roxΔ::kanMX**, PTEF1-SpyCas9-TCYC1, PTEF1-CroCPR-TPRM9, PPGK1-CroCYB5-TIDP1, PMLS1-AgrGPPS2-TVPS13, PFBA1-GgaFPSN144W-TIDP1, PPGK1-IDI1-TPRM9, PTDH3-tHMG1-TADH1, PICL1-ERG20F96W, N127WtCroGES-TCYC1, **PGAL1-RgnTDC**-TPRM9, PTDH3-CroG8H-TADH1, PPGK1-Vmi8HGO-A -TADH1,PFBA1-NcaISY-TCYC1, PTEF1-NcaMLPLA-TADH1, PTEF2-CroIO-TCYC1, PFBA1-CroADH2-TCPS1,PPGK1-Cro7DLGT-TVPS13, PTEF2-Cro7DLH-TCYC1, PTDH3-CroLAMT-TADH1, PTPI1-CroSLS-TIDP1,PFBA1-CroSTR-TPRM9,PTEF1-RseSGD-TADH1,PGK1-CroTHAS1-TCYC1, **PGAL2-CroTHAS1**-TCYC1, PTDH3-CroSS-TADH1	This study
ScH125	MAT**a**, his3D1, leu2-3_112, ura3-52, trp1-289, atf1Δ oye2Δ adh6Δ oye3Δ ari1Δ, PTEF1-SpyCas9-TCYC1, PTEF1-CroCPR-TPRM9, PPGK1-CroCYB5-TIDP1, PMLS1-AgrGPPS2-TVPS13, PFBA1-GgaFPSN144W-TIDP1, PPGK1-IDI1-TPRM9, PTDH3-tHMG1-TADH1, PICL1-ERG20F96W, N127WtCroGES-TCYC1, PGAL1-RgnTDC-TPRM9, PTDH3-CroG8H-TADH1, PPGK1-Vmi8HGO-A -TADH1,PFBA1-NcaISY-TCYC1, PTEF1-NcaMLPLA-TADH1, PTEF2-CroIO-TCYC1, PFBA1-CroADH2-TCPS1,PPGK1-Cro7DLGT-TVPS13, PTEF2-Cro7DLH-TCYC1, PTDH3-CroLAMT-TADH1, PTPI1-CroSLS-	This study

	TIDP1,PFBA1-CroSTR-TPRM9, PTEF1-RseSGD-TADH1, PGK1-CroHYS-TCYC1, PTDH3-CroSS-TADH1	
ScH132	MAT**a**, his3D1, leu2-3_112, ura3-52, trp1-289, atf1Δ oye2Δ adh6Δ oye3Δ ari1Δ, roxΔ::kanMX, PTEF1-SpyCas9-TCYC1, PTEF1-CroCPR-TPRM9, PPGK1-CroCYB5-TIDP1, PMLS1-AgrGPPS2-TVPS13, PFBA1-GgaFPSN144W-TIDP1, PPGK1-IDI1-TPRM9, PTDH3-tHMG1-TADH1, PICL1-ERG20F96W, N127WtCroGES-TCYC1, PGAL1-RgnTDC-TPRM9, PTDH3-CroG8H-TADH1, PPGK1-Vmi8HGO-A -TADH1,PFBA1-NcaISY-TCYC1, PTEF1-NcaMLPLA-TADH1, PTEF2-CroIO-TCYC1, PFBA1-CroADH2-TCPS1,PPGK1-Cro7DLGT-TVPS13, PTEF2-Cro7DLH-TCYC1, PTDH3-CroLAMT-TADH1, PTPI1-CroSLS-TIDP1,PFBA1-CroSTR-TPRM9,PTEF1-RseSGD-TADH1,PGK1-CroTHAS1-TCYC1, PGAL2-CroTHAS1-TCYC1, PTDH3-CroSS-TADH1 **PGAL1-LaeRebH-TADH1, PGAL2-ecoSsuE-TCYC1**	This study
ScH135	MAT**a**, his3D1, leu2-3_112, ura3-52, trp1-289, atf1Δ oye2Δ adh6Δ oye3Δ ari1Δ, roxΔ::kanMX, PTEF1-SpyCas9-TCYC1, PTEF1-CroCPR-TPRM9, PPGK1-CroCYB5-TIDP1, PMLS1-AgrGPPS2-TVPS13, PFBA1-GgaFPSN144W-TIDP1, PPGK1-IDI1-TPRM9, PTDH3-tHMG1-TADH1, PICL1-ERG20F96W, N127WtCroGES-TCYC1, PGAL1-RgnTDC-TPRM9, PTDH3-CroG8H-TADH1, PPGK1-Vmi8HGO-A -TADH1,PFBA1-NcaISY-TCYC1, PTEF1-NcaMLPLA-TADH1, PTEF2-CroIO-TCYC1, PFBA1-CroADH2-TCPS1,PPGK1-Cro7DLGT-TVPS13, PTEF2-Cro7DLH-TCYC1, PTDH3-CroLAMT-TADH1, PTPI1-CroSLS-TIDP1,PFBA1-CroSTR-TPRM9,PTEF1-RseSGD-TADH1,PGK1-CroTHAS1-TCYC1, PGAL2-CroTHAS1-TCYC1, PTDH3-CroSS-TADH1 **PGAL1-Trx_LaeRebH-TADH1, PGAL2-ecoSsuE-TCYC1**	This study
ScH137	MAT**a**, his3D1, leu2-3_112, ura3-52, trp1-289, atf1Δ oye2Δ adh6Δ oye3Δ ari1Δ, roxΔ::kanMX, PTEF1-SpyCas9-TCYC1, PTEF1-CroCPR-TPRM9, PPGK1-CroCYB5-TIDP1, PMLS1-AgrGPPS2-TVPS13, PFBA1-GgaFPSN144W-TIDP1, PPGK1-IDI1-TPRM9, PTDH3-tHMG1-TADH1, PICL1-ERG20F96W, N127WtCroGES-TCYC1, PGAL1-RgnTDC-TPRM9, PTDH3-CroG8H-TADH1, PPGK1-Vmi8HGO-A -TADH1,PFBA1-NcaISY-TCYC1, PTEF1-NcaMLPLA-TADH1, PTEF2-CroIO-TCYC1, PFBA1-CroADH2-TCPS1,PPGK1-Cro7DLGT-TVPS13, PTEF2-Cro7DLH-TCYC1, PTDH3-CroLAMT-TADH1, PTPI1-CroSLS-TIDP1,PFBA1-CroSTR-TPRM9,PTEF1-RseSGD-TADH1,PGK1-CroTHAS1-TCYC1, PGAL2-CroTHAS1-TCYC1, PTDH3-CroSS-TADH1 **PGAL1-T7B9_LaeRebH-TADH1, PGAL2-ecoSsuE-TCYC1**	This study
ScH143	MAT**a**, his3D1, leu2-3_112, ura3-52, trp1-289, atf1Δ oye2Δ adh6Δ oye3Δ ari1Δ, roxΔ::kanMX, PTEF1-SpyCas9-TCYC1, PTEF1-CroCPR-TPRM9, PPGK1-CroCYB5-TIDP1, PMLS1-AgrGPPS2-TVPS13, PFBA1-GgaFPSN144W-TIDP1, PPGK1-IDI1-TPRM9, PTDH3-tHMG1-TADH1, PICL1-ERG20F96W, N127WtCroGES-TCYC1, PGAL1-RgnTDC-TPRM9, PTDH3-CroG8H-TADH1, PPGK1-Vmi8HGO-A -TADH1,PFBA1-NcaISY-TCYC1, PTEF1-NcaMLPLA-TADH1, PTEF2-CroIO-TCYC1, PFBA1-CroADH2-TCPS1,PPGK1-Cro7DLGT-TVPS13, PTEF2-Cro7DLH-TCYC1, PTDH3-CroLAMT-TADH1, PTPI1-CroSLS-TIDP1,PFBA1-CroSTR-TPRM9,PTEF1-RseSGD-TADH1,PGK1-CroTHAS1-TCYC1, PGAL2-CroTHAS1-TCYC1, PTDH3-CroSS-TADH1 **PGAL1-LaeRebH-TADH1, PGAL1-LaeRebH-TADH1, PGAL1-LaeRebH-TADH1, PGAL2-ecoSsuE-TCYC1, PGAL2-ecoSsuE-TCYC1, PGAL2-ecoSsuE-TCYC1**	This study
ScH144	MAT**a**, his3D1, leu2-3_112, ura3-52, trp1-289, atf1Δ oye2Δ adh6Δ oye3Δ ari1Δ, **roxΔ::kanMX**, PTEF1-SpyCas9-TCYC1, PTEF1-CroCPR-TPRM9, PPGK1-CroCYB5-TIDP1, PMLS1PTEF2-AgrGPPS2-TVPS13, PFBA1-GgaFPSN144W-TIDP1, PPGK1-IDI1-TPRM9, PTDH3-tHMG1-TADH1, PICL1pCCW12-ERG20F96W, N127WtCroGES-TCYC1, PPGK1-CroTDC-TPRM9, PTDH3-CroG8H-TADH1, PPGK1-Vmi8HGO-A -TADH1,PFBA1-NcaISY-TCYC1, PTEF1-NcaMLPLA-TADH1, PTEF2-CroIO-TCYC1, PFBA1-CroADH2-TCPS1,PPGK1-Cro7DLGT-TVPS13,	This study

	PTEF2-Cro7DLH-TCYC1, PTDH3-CroLAMT-TADH1, PTPI1-CroSLS-TIDP1,PFBA1-CroSTR-TPRM9, PTEF1-RseSGD-TADH1,PGK1-CroTHAS1-TCYC1, PTDH3-CroSS_nat-TADH1	
LP72	MATα, his3D1; leu2-3_112; ura3-52; trp1-289; MAL2-8c; SUC2 +[LP1.T8]+ [LP2.T10]+ [LP3.T7] +[LP4.T9]	[17]
sDR023	MATα, his3D1; leu2-3_112; ura3-52; trp1-289; MAL2-8c; SUC2 +[LP1.T8] +[LP2.T10] +[LP3.T7:3x_yEGFP3] +[LP4.T9] +pcfb1767(TRP) +X-4: 4xUAS-SSA1p-365: mKate2	This study
sDR024	MATα, his3D1; leu2-3_112; ura3-52; trp1-289; MAL2-8c; SUC2 +[LP1.T8] +[LP2.T10] +[LP3.T7:3x_YFPm4] +[LP4.T9] +pcfb1767(TRP) +X-4: 4xUAS-SSA1p-365: mKate2 +HSP104:yEGFP3	This study
sDR028	MATα, his3D1; leu2-3_112; ura3-52; trp1-289; MAL2-8c; SUC2 +[LP1.T8] +[LP2.T10] +[LP3.T7:3x_RebH] +[LP4.T9] +pcfb1767(TRP) +X-4: 4xUAS-SSA1p-365: mKate2 +HSP104:yEGFP3	This study
scH122	MATa; his3D1; leu2-3_112; ura3-52; trp1-289; pTEF1-SpCas9-tCYC1, **PGAL1-LaeRebH-TADH1**, **PGAL2-ecoSsuE-TCYC1**	This study

Supplementary Table 6. Metabolite retention times (RTs) and quantifier and qualifier fragments used for Multiple Reaction Monitoring (MRM).

Metabolite	RT (min)	Molecular formula	Monoisotopic mass	$[M+H]^{+1}$	Quantifier (CE [V])	Qualifier (CE [V])
loganin	2.64	$C_{17}H_{26}O_{10}$	390.15	391.2	229.0 [7]	179 [17], 211 [7]
tryptamine	2.5	$C_{10}H_{12}N_2$	160.10	161.1	144.1 [9]	117 [28], 127 [27]
caffeine (IS)	2.58	$C_8H_{10}N_4O_2$	194.08	195.1	138.0 [15]	123 [28], 110 [22]
secologanin	2.9	$C_{17}H_{24}O_{10}$	388.14	389.1	227.2 [5]	107 [10], 165 [7]
strictosidine	3.11	$C_{27}H_{34}N_2O_9$	530.23	531.2	352.0 [25]	282 [30], 144 [35]
tetrahydro- alstonine	3.46	$C_{21}H_{24}N_2O_3$	352.18	353.2	144.0 [25]	222 [18], 210 [18]
ajmalicine	3.46	$C_{21}H_{24}N_2O_3$	352.18	353.2	144.0 [25]	222 [18], 210 [18]
alstonine	3.57	$C_{21}H_{20}N_2O_3$	348.147	349.1	262.9 [25],	316.9 [20], 234.9 [30]
serpentine	3.57	$C_{21}H_{20}N_2O_3$	348.147	349.1	262.9 [25],	316.9 [20], 234.9 [30]

Supplementary Table 7. Concentration of compounds used in analytical standards.

	tryptamine, loganin, secologanin, strictosidine (mg/L)	alstonine, serpentine (mg/L)	Tetrahydroalstonine, ajmalicine (mg/L)
Level-10	50	0.5	2
Level-9	25	0.25	1
Level-8	10	0.125	0.4
Level-7	5	0.05	0.2
Level-6	2.5	0.025	0.1
Level-5	1	0.0125	0.04
Level-4	0.5	0.005	0.02
Level-3	0.25	0.0025	0.01
Level-2	0.1	0.00125	0.004
Level-1	0.05	0.0005	0.002

Supplementary Table 8. Prior art and results from this study on promiscuity of MIA enzymes for halogenated substrate derivatives. This table tabulates the prior art that has tested the promiscuity of each MIA enzyme-substrate pair (columns) and halogen derivatisation (row, tryptophan atom number) in this study. The studies are color coded red (in vitro), green (in planta), black (yeast) or blue (this study). Where a cell appears gray, the promiscuity for that enzyme for this substrate has not been assessed.

Derivative (tryptophan carbon numbering)	*sce*TRP5 (indole)	*cro*TDC (tryptophan)	*rgn*TDC (tryptophan)	*cro*STR (tryptamine)	*rse*SGD (strictosidine)	*cro*THAS (strictosidine aglycone)	*cro*HYS (strictosidine aglycone)	*cro*SS (ajmalicine)	*croSS (THA)*
**4F**	This study	This study	This study<br>McDonald et al, 2019 [9]	This study<br>Schott et al, 2023 [18]<br>McCoy et al, 2006 [19]	This study<br>Schott et al, 2023[18]<br>McCoy et al, 2006[19]	This study	This study	This study	This study
**5F**	This study	This study	This study	This study<br>McCoy and O'Connor, 2006 [20]<br>Schott et al, 2023[18]<br>McCoy et al, 2006[19]	This study<br>McCoy et al, 2006[19]<br>McCoy and O'Connor, 2006[20]<br>Schott et al, 2023[18]<br>Yerkes et al, 2008 [21]	This study	This study<br>McCoy and O'Connor, 2006[20]	This study<br>McCoy and O'Connor, 2006[20]	This study
**6F**	This study	This study	This study	This study<br>McCoy and O'Connor, 2006[20]<br>Schott et al, 2023[18]<br>McCoy et al, 2006[19]	This study<br>McCoy et al, 2006[19]<br>McCoy and O'Connor, 2006[20]<br>Schott et al, 2023[18]<br>Yerkes et al, 2008 [21]	This study	This study<br>McCoy and O'Connor, 2006[20]	This study<br>McCoy and O'Connor, 2006[20]	This study

**7F**	This study	This study	This study	This study McCoy et al, 2006[19] Misa et al, 2022 [22] Shahsavarani et al, 2023 [23]	This study McCoy et al, 2006[19] Schott et al, 2023[18] Shahsavarani et al, 2023[23]	This study	This study	This study	This study
**4Cl**	This study	This study	This study McDonald et al, 2019[9]	This study					
**5Cl**	This study	This study Runguphan et al, 2010 [24] Milne et al. 2022 [25]	This study Runguphan and O'Connor, 2009 [26] McDonald et al, 2019[9]	This study Bernhardt et al, 2007[27] Runguphan and O'Connor, 2009 [26] Runguphan et al, 2010[24] Shahsavarani et al, 2023[23]	Runguphan et al, 2010[24] Bernhardt et al, 2007[27]		Bernhardt et al, 2007[27]		
**6Cl**	This study	This study Milne et al. 2022[25]	This study McDonald et al, 2019[9]	This study Misa et al, 2022[22]					
**7Cl**	This study	This study Runguphan et al, 2010[24]	This study McDonald et al, 2019[9]	This study Runguphan et al, 2010[24] Glenn et al, 2011 Misa et al, 2022[22]	This study Runguphan et al, 2010[24] Glenn et al, 2011 [28]	This study	This study	This study	This study
**4Br**	This study	This study	This study McDonald et al, 2019[9]	This study					
**5Br**	This study	This study Milne et al. 2022[25]	This study Runguphan and O'Connor, 2009 [26] McDonald et al, 2019[9]	This study Bernhardt et al, 2007 [27]Runguphan and O'Connor, 2009 [26] Misa et al, 2022[22]	Bernhardt et al, 2007[27]		Bernhardt et al, 2007[27]		
**6Br**	This study	This study Milne et al. 2022[25]	This study McDonald et al, 2019[9]	This study					
**7Br**	This study	This study	This study McDonald et al, 2019[9]	This study Runguphan et al, 2010[24]	This study	This study	This study	This study	This study
**4,5diF**	This study	This study	This study	This study	This study		This study	This study	
**4,6diF**	This study	This study	This study	This study	This study		This study	This study	

**4,7diF**	This study	This study	This study	This study	This study		This study	This study	
**5,6diF**	This study	This study	This study	This study	This study	This study	This study	This study	This study
**5,7diF**	This study	This study	This study	This study	This study		This study	This study	
**6,7diF**	This study	This study	This study	This study	This study	This study	This study	This study	This study

Supplementary Table 9. Metabolite retention times, masses and identifying fragments used in HRMS.

Compound	Formula	RT (Min)	Theoretical Monoisotopic mass	Theoretical [M+H]+1	Observed [M+H]+1	ppm	MS-MS Identifier Peaks
Secologanin	C17H24O10	5.9	388.1369	389.1447	389.1446	-0.3	107.04, 165.05
Tryptophan	C11H12N2O2	4.8	204.0898	205.0976	205.0971	-2.4	118.06, 146.05, 188.07
Tryptamine	C10H12N2	5.2	160.1	161.1078	161.1072	-3.7	144.08
Strictosidine	C27H34N2O9	6.4	530.2264	531.2342	531.2340	-0.4	144.08, 514.20
Tetrahydroalstonine	C21H24N2O3	6.8	352.1787	353.1865	353.1862	-0.8	144.08
Ajmalicine	C21H24N2O3	6.8	352.1787	353.1865	353.1862	-0.8	144.08
Alstonine	C21H20N2O3	6.9	348.1474	349.1552	349.1550	-0.6	349.15, 263.08, 235.08

Serpentine	C21H20N2O3	6.8	348.1474	349.1552	349.1550	-0.6	349.15, 263.08, 235.08
Fluorotryptophan	FC11H11N2O2	4F: 5.3, 5F: 5.3, 6F: 5.2, 7F: 5.3	222.0805	223.0883	223.0877	-2.7	136.05, 164.05, 206.06
Fluorotryptamine	FC10H11N2	4F: 5.7, 5F: 5.6, 6F: 5.5, 7F: 5.6	178.0906	179.0984	179.0978	-3.4	162.07
Fluorostrictosidine	FC27H33N2O9	4F: 6.5, 5F: 6.5, 6F: 6.5, 7F: 6.5	548.217	549.2248	549.2250	0.4	Not observed
Fluorotetrahydroalstonine	FC21H23N2O3	Not observed	370.1693	371.1771	Not observed	Not observed	Not observed
Fluoroajmalicine	FC21H23N2O3	Not observed	370.1693	371.1771	Not observed	Not observed	Not observed
Fluoroalstonine	FC21H19N2O3	4F: 7.0, 5F: 6.9, 6F: 6.9, 7F: 6.9	366.138	367.1458	367.1454	-1.1	153.07, 281.07, 367.14
Fluoroserpentine	FC21H19N2O3	4F: 6.9, 5F: 6.9, 6F: 6.9, 7F: 6.8	366.138	367.1458	367.1454	-1.1	153.07, 281.07, 367.14
Chlorotryptophan	ClC11H11N2O2	4Cl: 5.8, 5Cl: 5.8, 6Cl: 5.9, 7Cl: 5.8	238.0509	239.0587	239.0582	-2.1	152.02, 180.02, 222.03

Chlorotryptamine	ClC10H11N2	4Cl: 6.1, 5Cl: 6.1, 6Cl: 6.2, 7Cl: 6.1	194.0611	195.0689	195.0684	-2.6	178.04
Chlorostrictosidine	ClC27H33N2O9	4Cl: 6.8, 7Cl: 6.7	564.1874	565.1952	565.1938	-2.5	178.04, 548.16
Chlorotetrahydroalstonine	ClC21H23N2O3	Not observed	386.1397	387.1475	Not observed	Not observed	Not observed
Chloroajmalicine	ClC21H23N2O3	Not observed	386.1397	387.1475	Not observed	Not observed	Not observed
Chloroalstonine	ClC21H19N2O3	7Cl: 7.0	382.1084	383.1162	383.1159	-0.8	241.05, 297.04, 383.11
Chloroserpentine	ClC21H19N2O3	7Cl: 7.1	382.1084	383.1162	383.1159	-0.8	241.05, 297.04, 383.11
Bromotryptophan	BrC11H11N2O2	Not observed	282.0004	283.0082	283.0077	-1.8	195.97, 223.97, 265.98
Bromotryptamine	BrC10H11N2	4Br: 6.2, 5Br: 6.3, 6Br: 6.3, 7Br: 6.2	238.0106	239.0184	239.0178	-2.5	221.99
Bromostrictosidine	BrC27H33N2O9	Not confirmed	608.1369	609.1447	Not confirmed	Not confirmed	Not confirmed
Bromotetrahydroalstonine	BrC21H23N2O3	Not observed	430.0892	431.097	Not observed	Not observed	Not observed

Bromoajmalicine	BrC21H23N2O3	Not observed	430.0892	431.097	Not observed	Not observed	Not observed
Bromoalstonine	BrC21H19N2O3	7Br: 7.2	426.0579	427.0657	427.0654	-0.7	312.99, 340.99, 427.06
Bromoserpentine	BrC21H19N2O3	7Br: 7.2	426.0579	427.0657	427.0654	-0.7	312.99, 340.99, 427.06
Difluorotryptophan	F2C11H10N2O2	4,5F: 5.6, 4,6F: 5.6, 4,7F: 5.5, 5,6F: 5.6, 5,7F: 5.6, 6,7F: 5.6,	240.071	241.0788	241.0783	-2.1	154.04, 182.04, 224.05
Difluorotryptamine	F2C10H10N2	4,5F: 5.9, 4,6F: 5.8, 4,7F: 5.9, 5,6F: 5.9, 5,7F: 5.9, 6,7F: 5.9	196.0812	197.089	197.0885	-2.5	180.06
Difluorostrictosidine	F2C27H32N2O9	4,5F: 6.6, 4,6F: 6.7, 4,7F: 6.6	566.2075	567.2153	567.2149	-0.7	180.06, 548.17
Difluorotetrahydroalstonine	F2C21H22N2O3	Not observed	388.1598	389.1676	Not observed	Not observed	Not observed
Difluoroajmalicine	F2C21H22N2O3	Not observed	388.1598	389.1676	Not observed	Not observed	Not observed
Difluoroalstonine	F2C21H18N2O3	5,6F: 7.1, 6,7F: 7.0,	384.1285	385.1363	385.1361	-0.5	243.07, 299.06, 385.13
Difluoroserpentine	F2C21H18N2O3	4,5F: 7.0, 4,6F: 7.0, 4,7F: 6.9, 5,7F: 6.9, 6,7F: 7.0,	384.1285	385.1363	385.1361	-0.5	243.07, 299.06, 385.13

References

1. Zhang, J. *et al.* A microbial supply chain for production of the anti-cancer drug vinblastine. *Nature* **609**, 341–347 (2022).
2. Dang, T.-T. T. *et al.* Sarpagan bridge enzyme has substrate-controlled cyclization and aromatization modes. *Nat. Chem. Biol.* **14**, 760–763 (2018).
3. Stavrinides, A. *et al.* Structural investigation of heteroyohimbine alkaloid synthesis reveals active site elements that control stereoselectivity. *Nat. Commun.* **7**, 12116 (2016).
4. Stavrinides, A. *et al.* Unlocking the diversity of alkaloids in Catharanthus roseus: nuclear localization suggests metabolic channeling in secondary metabolism. *Chem. Biol.* **22**, 336–341 (2015).
5. Gerasimenko, I., Sheludko, Y., Ma, X. & Stöckigt, J. Heterologous expression of a Rauvolfia cDNA encoding strictosidine glucosidase, a biosynthetic key to over 2000 monoterpenoid indole alkaloids. *Eur. J. Biochem.* **269**, 2204–2213 (2002).
6. Yamamoto, K. *et al.* Improved virus-induced gene silencing allows discovery of a serpentine synthase gene in Catharanthus roseus. *Plant Physiol.* **187**, 846–857 (2021).
7. Yeh, E., Garneau, S. & Walsh, C. T. Robust in vitro activity of RebF and RebH, a two-component reductase/halogenase, generating 7-chlorotryptophan during rebeccamycin biosynthesis. *Proc Natl Acad Sci USA* **102**, 3960–3965 (2005).
8. Heemstra, J. R. & Walsh, C. T. Tandem action of the O2- and FADH2-dependent halogenases KtzQ and KtzR produce 6,7-dichlorotryptophan for kutzneride assembly. *J. Am. Chem. Soc.* **130**, 14024–14025 (2008).
9. McDonald, A. D., Perkins, L. J. & Buller, A. R. Facile in vitro biocatalytic production of

diverse tryptamines. *Chembiochem* **20**, 1939–1944 (2019).

10. LaVallie, E. R. *et al.* A thioredoxin gene fusion expression system that circumvents inclusion body formation in the E. coli cytoplasm. *Biotechnology (NY)* **11**, 187–193 (1993).
11. Manca, A. *et al.* Detailed physical analysis of a 1.5-megabase YAC contig containing the MXI1 and ADRA2A genes. *Genomics* **45**, 407–411 (1997).
12. England, C. G., Ehlerding, E. B. & Cai, W. Nanoluc: A small luciferase is brightening up the field of bioluminescence. *Bioconjug. Chem.* **27**, 1175–1187 (2016).
13. Jensen, N. B. *et al.* EasyClone: method for iterative chromosomal integration of multiple genes in Saccharomyces cerevisiae. *FEMS Yeast Res.* **14**, 238–248 (2014).
14. DiCarlo, J. E. *et al.* Genome engineering in Saccharomyces cerevisiae using CRISPR-Cas systems. *Nucleic Acids Res.* **41**, 4336–4343 (2013).
15. Romero-Suarez, D. *et al.* A reporter system for cytosolic protein aggregates in yeast. *ACS Synth. Biol.* **10**, 466–477 (2021).
16. Shaw, W. M. *et al.* Engineering a model cell for rational tuning of GPCR signaling. *Cell* **177**, 782-796.e27 (2019).
17. Bourgeois, L., Pyne, M. E. & Martin, V. J. J. A highly characterized synthetic landing pad system for precise multicopy gene integration in yeast. *ACS Synth. Biol.* **7**, 2675–2685 (2018).
18. Schotte, C. *et al.* Directed biosynthesis of mitragynine stereoisomers. *J. Am. Chem. Soc.* (2023) doi:10.1021/jacs.2c13644.
19. McCoy, E., Galan, M. C. & O'Connor, S. E. Substrate specificity of strictosidine synthase. *Bioorg. Med. Chem. Lett.* **16**, 2475–2478 (2006).
20. McCoy, E. & O'Connor, S. E. Directed biosynthesis of alkaloid analogs in the medicinal plant Catharanthus roseus. *J. Am. Chem. Soc.* **128**, 14276–14277 (2006).
21. Yerkes, N. *et al.* Substrate specificity and diastereoselectivity of strictosidine glucosidase, a key enzyme in monoterpene indole alkaloid biosynthesis. *Bioorg. Med. Chem. Lett.* **18**,

3095–3098 (2008).

22. Misa, J., Billingsley, J. M., Niwa, K., Yu, R. K. & Tang, Y. Engineered production of strictosidine and analogues in yeast. *ACS Synth. Biol.* (2022) doi:10.1021/acssynbio.2c00037.

23. Shahsavarani, M. *et al.* Improved protein glycosylation enabled heterologous biosynthesis of monoterpenoid indole alkaloids and their unnatural derivatives in yeast. *Metab. Eng. Commun.* **16**, e00215 (2023).

24. Runguphan, W., Qu, X. & O'Connor, S. E. Integrating carbon-halogen bond formation into medicinal plant metabolism. *Nature* **468**, 461–464 (2010).

25. Milne, N. *et al.* Metabolic engineering of Saccharomyces cerevisiae for the de novo production of psilocybin and related tryptamine derivatives. *Metab. Eng.* **60**, 25–36 (2020).

26. Runguphan, W. & O'Connor, S. E. Metabolic reprogramming of periwinkle plant culture. *Nat. Chem. Biol.* **5**, 151–153 (2009).

27. Bernhardt, P., McCoy, E. & O'Connor, S. E. Rapid identification of enzyme variants for reengineered alkaloid biosynthesis in periwinkle. *Chem. Biol.* **14**, 888–897 (2007).

28. Glenn, W. S., Nims, E. & O'Connor, S. E. Reengineering a tryptophan halogenase to preferentially chlorinate a direct alkaloid precursor. *J. Am. Chem. Soc.* **133**, 19346–19349 (2011).
