# Supplementary material for: Biosynthesis of natural and halogenated plant monoterpene indole alkaloids in yeast
Source: Nat Chem Biol. 2023 Nov 6;19(12):1551–60. doi: 10.1038/s41589-023-01430-2 (PMC10667104; doi:10.1038/s41589-023-01430-2)
Supplement: Supplementary file 2 — Reporting Summary [file 41589_2023_1430_MOESM2_ESM.pdf]

nature portfolio

Corresponding author(s): Michael Krogh Jensen
Jie Zhang

Last updated by author(s): May 15, 2023

Reporting Summary

Nature Portfolio wishes to improve the reproducibility of the work that we publish. This form provides structure for consistency and transparency in reporting. For further information on Nature Portfolio policies, see our Editorial Policies and the Editorial Policy Checklist.

Statistics

For all statistical analyses, confirm that the following items are present in the figure legend, table legend, main text, or Methods section.

n/a	Confirmed	
☐	☒	The exact sample size (*n*) for each experimental group/condition, given as a discrete number and unit of measurement
☐	☒	A statement on whether measurements were taken from distinct samples or whether the same sample was measured repeatedly
☐	☒	The statistical test(s) used AND whether they are one- or two-sided<br>*Only common tests should be described solely by name; describe more complex techniques in the Methods section.*
☒	☐	A description of all covariates tested
☒	☐	A description of any assumptions or corrections, such as tests of normality and adjustment for multiple comparisons
☐	☒	A full description of the statistical parameters including central tendency (e.g. means) or other basic estimates (e.g. regression coefficient) AND variation (e.g. standard deviation) or associated estimates of uncertainty (e.g. confidence intervals)
☐	☒	For null hypothesis testing, the test statistic (e.g. *F*, *t*, *r*) with confidence intervals, effect sizes, degrees of freedom and *P* value noted<br>*Give P values as exact values whenever suitable.*
☒	☐	For Bayesian analysis, information on the choice of priors and Markov chain Monte Carlo settings
☒	☐	For hierarchical and complex designs, identification of the appropriate level for tests and full reporting of outcomes
☒	☐	Estimates of effect sizes (e.g. Cohen's *d*, Pearson's *r*), indicating how they were calculated

Our web collection on statistics for biologists contains articles on many of the points above.

Software and code

Policy information about availability of computer code

Data collection	No software was used for data collection
Data analysis	Freestyle 1.6<br>Xcalibur 4.2.28.24<br>Python 3.11.2<br>Bruker MS Workstation version 8.210.0.253<br>FlowJo 10.8.1

For manuscripts utilizing custom algorithms or software that are central to the research but not yet described in published literature, software must be made available to editors and reviewers. We strongly encourage code deposition in a community repository (e.g. GitHub). See the Nature Portfolio guidelines for submitting code & software for further information.

Data

Policy information about availability of data

All manuscripts must include a data availability statement. This statement should provide the following information, where applicable:

- Accession codes, unique identifiers, or web links for publicly available datasets
- A description of any restrictions on data availability
- For clinical datasets or third party data, please ensure that the statement adheres to our policy

Data supporting the findings of this work are available within the paper and its Supplementary Information files. A reporting summary for this article is available as a Supplementary Information file. The datasets generated and analyzed during the current study are available from the corresponding author upon request.
There are no restrictions on data availability when the study is published.

Field-specific reporting

Please select the one below that is the best fit for your research. If you are not sure, read the appropriate sections before making your selection.

☒ Life sciences ☐ Behavioural & social sciences ☐ Ecological, evolutionary & environmental sciences

For a reference copy of the document with all sections, see nature.com/documents/nr-reporting-summary-flat.pdf

Life sciences study design

All studies must disclose on these points even when the disclosure is negative.

Sample size	No sample size calculation was performed. n =3 was deemed sufficient, as results were highly reproducible between replicates.
Data exclusions	No data was excluded.
Replication	All data replicated with n >= 3 apart for results presented in Fig. 2b where n=2
Randomization	For all reported strain designs, colonies were randomly picked from transformation plates.
Blinding	Beyond randomization of colony picking no blinding was needed in this study, because the work involves rationally engineered strains.

Reporting for specific materials, systems and methods

We require information from authors about some types of materials, experimental systems and methods used in many studies. Here, indicate whether each material, system or method listed is relevant to your study. If you are not sure if a list item applies to your research, read the appropriate section before selecting a response.

Materials & experimental systems

n/a	Involved in the study	
☒	☐	Antibodies
☐	☒	Eukaryotic cell lines
☒	☐	Palaeontology and archaeology
☒	☐	Animals and other organisms
☒	☐	Human research participants
☒	☐	Clinical data
☒	☐	Dual use research of concern

Methods

n/a	Involved in the study	
☒	☐	ChIP-seq
☐	☒	Flow cytometry
☒	☐	MRI-based neuroimaging

Eukaryotic cell lines

Policy information about cell lines

Cell line source(s)	For the expression of the GPCR 5HT2C, we used Cos7 cells.
Authentication	Cos7 is an African green monkey kidney fibroblast-like cell line suitable for transfection and expression of human GPCRs. The cell line was originally obtained from the European Collection of Authenticated Cell Cultures (ECACC).
Mycoplasma contamination	The authors hereby confirm that the Cos7 cell line was tested negative for mycoplasm contamination.
Commonly misidentified lines (See ICLAC register)	We made use of no commonly misidentified cell lines

Flow Cytometry

Plots

Confirm that:

☒ The axis labels state the marker and fluorochrome used (e.g. CD4-FITC).

☒ The axis scales are clearly visible. Include numbers along axes only for bottom left plot of group (a 'group' is an analysis of identical markers).

☒ All plots are contour plots with outliers or pseudocolor plots.

☒ A numerical value for number of cells or percentage (with statistics) is provided.

Methodology

Sample preparation	Yeast cultures (Protocol for growing yeast cultures in Materials and Methods) in SC+2% Glucose or SC+2% Galactose in 96 well microtiter plates were loaded to the flow cytometer immmediately after incubation time was finished . We aimed to OD600= 0.2 measued in Synergy Mx (BioTek) and diluted cultures in PBS1x when necessary.
Instrument	MACSQuant Analyzer VYB Flow Cytometer (Miltenyi Biotec)
Software	FlowJo™ Software version 10.8.1 (BD)
Cell population abundance	Cell populations used for analyses contained singlet 4000 events per replicate
Gating strategy	All flow cytometry data was extracted as FCS files and gated in FlowLogic™ (Inivai Technologies). SSC-A, FSC-A, and fluorescence data points were derived from median values and median fluorescence intensities (MFI) of gated populations. Normalized MFI (nMFI) was obtained by normalizing to the mean of background MFIs. For statistics and data analysis means of medians and MFIs were applied ± standard deviation. Population proportions were derived directly from event counts per gate. For biosensor assays, small non-responsive cells were removed by consistent exclusive gates based on a minimum FSC-A values.

☒ Tick this box to confirm that a figure exemplifying the gating strategy is provided in the Supplementary Information.
